# Interactions of Both Pathogenic and Nonpathogenic CUG Clade *Candida* Species with Macrophages Share a Conserved Transcriptional Landscape

**DOI:** 10.1128/mbio.03317-21

**Published:** 2021-12-14

**Authors:** Andrew W. Pountain, John R. Collette, William M. Farrell, Michael C. Lorenz

**Affiliations:** a Department of Microbiology & Molecular Genetics, University of Texas Health Science Center at Houston, Houston, Texas, USA; b Department of Pathology and Immunology, Baylor College of Medicine, Houston, Texas, USA; Universidade de Sao Paulo

**Keywords:** *Candida*, host-pathogen interactions, macrophages, transcriptomics

## Abstract

*Candida* species are a leading cause of opportunistic, hospital-associated bloodstream infections with high mortality rates, typically in immunocompromised patients. Several species, including Candida albicans, the most prevalent cause of infection, belong to the monophyletic CUG clade of yeasts. Innate immune cells such as macrophages are crucial for controlling infection, and C. albicans responds to phagocytosis by a coordinated induction of pathways involved in catabolism of nonglucose carbon sources, termed alternative carbon metabolism, which together are essential for virulence. However, the interactions of other CUG clade species with macrophages have not been characterized. Here, we analyzed transcriptional responses to macrophage phagocytosis by six *Candida* species across a range of virulence and clinical importance. We define a core induced response common to pathogenic and nonpathogenic species alike, heavily weighted to alternative carbon metabolism. One prominent pathogen, Candida parapsilosis, showed species-specific expansion of phagocytosis-responsive genes, particularly metabolite transporters. C. albicans and Candida tropicalis, the other prominent pathogens, also had species-specific responses, but these were largely comprised of functionally uncharacterized genes. Transcriptional analysis of macrophages also demonstrated highly correlated proinflammatory transcriptional responses to different *Candida* species that were largely independent of fungal viability, suggesting that this response is driven by recognition of conserved cell wall components. This study significantly broadens our understanding of host interactions in CUG clade species, demonstrating that although metabolic plasticity is crucial for virulence in *Candida*, it alone is not sufficient to confer pathogenicity. Instead, we identify sets of mostly uncharacterized genes that may explain the evolution of pathogenicity.

## INTRODUCTION

Pathogenic yeasts of the genus *Candida* impose a huge burden on human health. Infections range from common superficial and nonlethal manifestations such as oral and vulvovaginal candidiasis to serious disseminated hematogenous and invasive forms of the disease, which affect an estimated 700,000 annually with a mortality rate of around 40% (1). Disseminated candidiasis is generally limited to immunocompromised individuals, the population of which has increased due to advances in medical interventions, e.g., organ transplantation, chemotherapy, and intravenous catheters (2). As disseminated candidiasis causes high mortality and prolonged hospitalization in this patient population, even with the use of antifungal drugs, understanding the pathogenic mechanisms of *Candida* species is an important priority.

**FIG 1:**
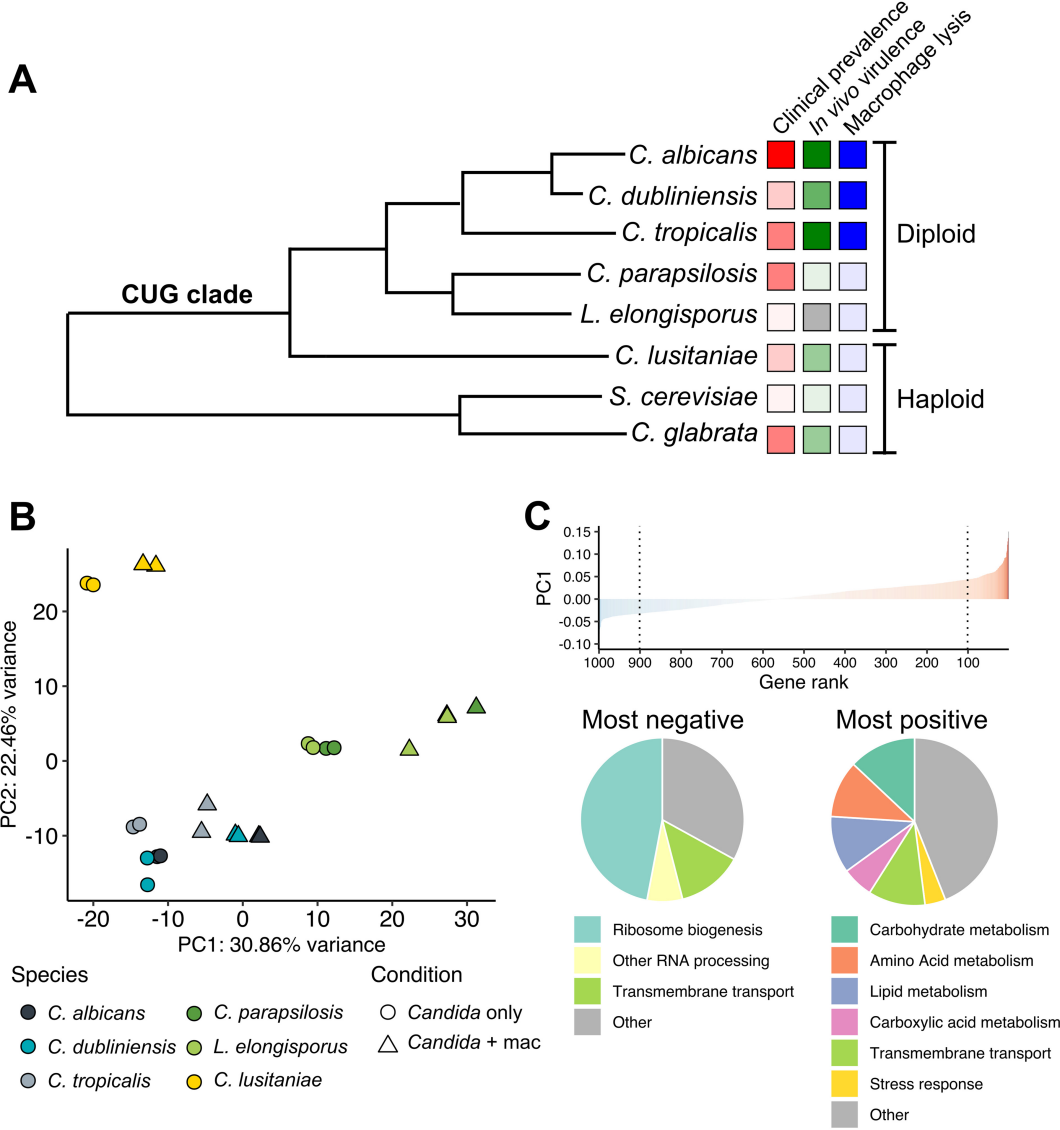
CUG clade *Candida* species exhibit a conserved response to phagocytosis. (A) The relationship of the CUG clade species characterized in this study, as well as two non-CUG clade species, is depicted as adapted from a previously described phylogenetic tree (13). Virulence-related phenotypes are indicated by colored boxes, with darker colors indicating more virulent/prevalent. Comparisons are strictly qualitative. Data are from references 2, 9, 12, 33, 96, and 97. (B) Principal-component analysis of mean-centered, log-transformed mapped fragment counts of orthologous groups of genes across species. The 1,000 genes with the highest variance in transformed counts were selected for analysis. (C) The same gene set used in panel B ranked by the loading of principal component (PC) 1. The 10% most negatively (left) and positively (right) contributing genes were analyzed for functional groupings as depicted in the pie charts for repressed (left) and induced (right) genes.

About 95% of infections are caused by just four species: Candida albicans, Candida glabrata, Candida parapsilosis, and Candida tropicalis (3, 4). Of these, all but C. glabrata are within the “CUG” clade (or CUG-Ser1) of fungi (Fig. 1A), in which CUG encodes serine instead of leucine (5–8). CUG clade species are believed to originate from a single ancestor (6). Thus, the CUG clade better describes the evolutionary relationships than the genus, since there are both CUG and non-CUG *Candida* species. Species in this clade show a spectrum of pathogenicity, with virulence in mouse models partially correlating with clinical prevalence (9, 10) (Fig. 1A) (also see reference 7). C. albicans is tightly, and perhaps obligately, associated with the mucosae of warm-blooded animals, especially humans. It is the most common cause of infection, though non-*albicans* species have increased in incidence (11) and more attention needs to be dedicated to their virulence traits and host interactions. C. tropicalis is less common clinically, but is as lethal as C. albicans in mouse models of disseminated disease (9, 10). In contrast, several other CUG clade species show reduced virulence: Candida lusitaniae (also known as Clavispora lusitaniae) and C. parapsilosis persist in the organs of infected mice but are not lethal (9, 10), and Candida dubliniensis, despite being the closest relative of C. albicans, is only rarely a cause of disseminated infection (3, 12). Of these, only C. parapsilosis is a major cause of candidiasis. Finally, species such as Lodderomyces elongisporus are almost never observed to cause infection. Thus, there is a spectrum of clinical incidence and virulence potential among CUG clade species.

The evolutionary relationship of CUG clade species supports a comparative approach to understand the basis of variable pathogenicity in this group. Comparative genomics reveals that clinically prevalent species show expansion of several gene families including cell wall proteins, metabolite transporters, and lipases (13), but differences in gene expression and the acquisition of novel gene functions must also underlie the differences in pathogenic potential.

The interaction of C. albicans with macrophages has been studied extensively (14–16) as a relevant and facile model of host-pathogen interactions, typified by a dynamic response that includes transcriptional, metabolic, and physiological changes, including the induction of filamentous growth that contributes to the eventual destruction of the phagocyte. A hallmark of the transcriptional response is a broad induction of genes associated with metabolism of nonglucose carbon sources, including amino acids, organic acids, and lipids, collectively termed alternative carbon metabolism (17, 18), implying that the phagolysosome is glucose poor. Consistent with this, these metabolic pathways are required for survival within macrophages, as well as virulence in mouse models (19–23). C. glabrata, the most common non-CUG clade pathogen, is similarly able to remodel its metabolism in response to phagocytosis (24), whereas its close nonpathogenic relative, Saccharomyces cerevisiae, exhibits a much less robust response (17). Within the CUG clade, however, the degree to which this metabolic response to phagocytosis is conserved is currently unknown.

Macrophages respond to fungi through a series of receptors such as dectin-1, which recognizes β-glucan in yeast cell walls (25, 26), and dectin-2, which binds α-mannans in both yeast and hyphae (27, 28). This recognition induces a strong proinflammatory transcriptional response, including increased expression of cytokine and chemokine genes (18, 29). Phagocytosis of C. albicans eventually activates the NLRP-3 inflammasome and pyroptosis (30, 31). Hyphal morphogenesis contributes to, but is not strictly required for, the induction of pyroptosis (32). Only C. albicans, C. dubliniensis, and C. tropicalis rapidly induce pyroptosis (33) (although C. parapsilosis may do so over much longer time scales [34]), and macrophage responses when confronted with other CUG clade species are not known.

We report here comparative analyses of the transcriptional responses of different *Candida* species and macrophages during phagocytosis. We find that the core metabolic response in C. albicans is conserved across the virulence spectrum. Thus, while this metabolic response is required for full virulence, it is not, in itself, sufficient to explain the difference in pathogenic potential between these species. There are also species-specific responses, and genes with higher expression in macrophages in C. albicans than other species are highly enriched for functions related to biofilm formation and hyphal growth. In contrast, macrophages do not significantly distinguish between *Candida* species, with nearly identical responses to both pathogens and nonpathogens alike at early time points, suggesting that this is driven by recognition of conserved fungal epitopes and cytokine signaling rather than fungal activity or metabolism. This work extends our understanding of the critical interaction between *Candida* species and innate immunity.

## RESULTS

### Transcriptional induction of alternative carbon metabolism upon phagocytosis is conserved in CUG clade species.

In order to compare responses of different *Candida* spp. across a range of virulence phenotypes within the CUG clade (Fig. 1A), we exposed *Candida* either to primary murine bone marrow-derived macrophages for 1 h, by which time the vast majority of fungal cells had been engulfed by macrophages, or to incubation in mammalian growth medium alone. At this point, there were no apparent differences in fungal or macrophage viability and C. albicans had only just begun hyphal growth. We were unable to isolate high-quality RNA from both the macrophage and fungus at the same time, so we split the cultures and used separate protocols for fungal or mouse RNA. In the fungal RNA, deep sequencing allowed detection of >96% of genes in all samples (Table 1; see also Fig. S1A in the supplemental material). Interreplicate correlation was excellent, indicating good reproducibility (Table 1; Fig. S1B). Pairwise differential expression analysis in each species showed broad responses to phagocytosis in each species (Table S1).

**TABLE 1:** Summary statistics for RNA sequencing data^*a*^

Species	No. of reads (million)	% mapped	% fungal	% detected	% detected (>10)	Cor (±mac)
C. albicans	16.8–23.3	99.0–99.1	97.1–99.0	95.4–96.8	89.8–92.2	1.00/1.00
C. dubliniensis	15.5–22.9	98.3–99.0	94.8–99.0	97.9–99.3	92.3–95.7	1.00/0.94
C. tropicalis	15.3–24.8	92.9–97.8	83.4–97.8	96.1–98.2	90.1–93.6	0.94/1.00
C. parapsilosis	15.3–20.5	99.2–99.5	98.1–99.3	99.1–99.5	96.1–97.6	0.99/0.99
*L. elongisporus*	14.2–57.8	98.0–98.4	96.5–98.3	97.3–98.5	92.0–96.1	0.93/0.99
C. lusitaniae	15.1–20.0	99.0–99.0	95.3–99.0	96.9–98.1	90.2–93.1	0.99/1.00

a For each species, number of reads passing quality filters, the percentage mapped to mouse or fungal genomes, the percentage mapped to fungal genomes, and the percentage of genes detected by at least one or 10 reads are shown, with intervals indicating range across samples. Spearman correlation coefficients of transcript abundance between replicates for each species and each condition (with or without macrophages) [Cor (±mac)] are also provided, with all intersample correlations provided in Fig. S1B.

10.1128/mbio.03317-21.1 FIG S1 Sample mapping statistics and intersample correlations. (A) Mapping statistics for fungal RNA-seq data. Adapter-trimmed reads were aligned to concatenated mouse-*Candida* genomes as described in Materials and Methods. Reads that mapped concordantly to mouse or *Candida* genomes or not concordantly to either are shown both as number of reads (left) and as percentage of total reads (right). (B) Pairwise Spearman correlations in transcript abundance between samples. Spearman correlation coefficients were calculated using log-transformed fragment counts for each sample-sample pair. Samples were clustered according to distance (1 − correlation coefficient). (C) Mapping statistics for macrophage RNA-seq data, displayed as in panel A. Download FIG S1, TIF file, 2.3 MB. Copyright © 2021 Pountain et al. 2021 Pountain et al. https://creativecommons.org/licenses/by/4.0/ This content is distributed under the terms of the Creative Commons Attribution 4.0 International license.

10.1128/mbio.03317-21.7 TABLE S1 Pairwise differential expression analysis, identification of core conserved responses to phagocytosis, and normalized count estimates for principal-component analysis. Pairwise differential expression analysis of the effect of phagocytosis was performed independently for each species using DESeq2. baseMean is the average expression across all samples, log_2_ fold change represents expression in macrophages divided by *Candida*-only samples with lfcSE representing error. Raw (pvalue) and multiple comparisons-adjusted *P* values (padj) are also provided. For the 92 genes induced and seven genes repressed in at least five out of six species (core induced and core repressed genes), the C. albicans gene ID, gene name, and description are shown. For induced genes, the “Mig1/Mig2 regulated” column indicates whether the gene is (“y”) or is not (“n”) within the Mig1/Mig2 regulon as defined in reference 39. Finally, in the tab “C. paraps versus L. elongis,” differential expression analysis was performed to determine the differentially phagocytosis-responsive genes between C. parapsilosis and *L. elongisporus*, as described in Materials and Methods. log2FoldChange is equivalent to the log-transformed fold change in fold changes between *L. elongisporus* and C. parapsilosis, and pvalue and padj indicate the raw and multiple comparisons-corrected *P* values for the significance of the interaction term between condition (*Candida* only or phagocytosed *Candida*) and species. A positive fold change indicates a higher degree of induction (more positive log_2_ fold change) in *L. elongisporus*, and a negative one indicates a higher degree of induction in C. parapsilosis. The second-to-last tab contains regularized, log-transformed abundance estimates for 4,376 conserved genes were obtained as described in Materials and Methods. For each group of orthologous genes, abundance estimates are shown for each sample along with gene IDs for each species. These were used for principal-component analysis as described for Fig. 1B on the 1,000 conserved genes with the highest variance in gene expression across samples. For each of these, identified by the C. albicans gene ID, the loadings for PC1 and PC2 are shown in the final tab along with C. albicans gene descriptions and names. Download Table S1, XLSX file, 5.4 MB. Copyright © 2021 Pountain et al. 2021 Pountain et al. https://creativecommons.org/licenses/by/4.0/ This content is distributed under the terms of the Creative Commons Attribution 4.0 International license.

To compare transcriptomes across *Candida* spp., we identified orthologous groups of genes using the *Candida* Gene Order Browser (CGOB) (35), which defines orthologs on the basis of both sequence homology and genomic synteny. Across the six species included, this gave a core set of 4,376 conserved genes, for which we obtained gene-level estimates of expression (Table S1; see Materials and Methods). Expression of orthologs across species showed a strong correlation (Spearman’s *r* between samples from different species, 0.78 to 0.96 [Fig. S1B]), suggesting broad conservation of gene expression within this set.

Using these estimates of expression of orthologous genes, we investigated global trends across the data using two methodologies. First, principal-component analysis (PCA) revealed both expression differences that followed phylogenetic groupings (PC2) and globally conserved responses, seen as a positive shift in PC1 upon exposure to macrophages (Fig. 1B). The 10% of genes that contribute most negatively to PC1 (those showing reduced expression in response to phagocytosis) were largely involved in ribosome biogenesis and RNA processing (Fig. 1C and Table S1). In contrast, the 10% of genes that most positively contributed to PC1 (those induced in response to phagocytosis) included a large number of genes involved in alternative carbon metabolism and metabolite transport. This is consistent with previous reports that C. albicans undergoes a significant change in metabolism, indicative of carbon starvation, upon phagocytosis (17, 18, 36). Notably, C. parapsilosis and *L. elongisporus* showed a strong positive shift along PC1 irrespective of condition (Fig. 1B), indicating that many genes strongly contributing to PC1 (e.g., alternative carbon metabolism) also had higher basal expression in these species.

**FIG 2:**
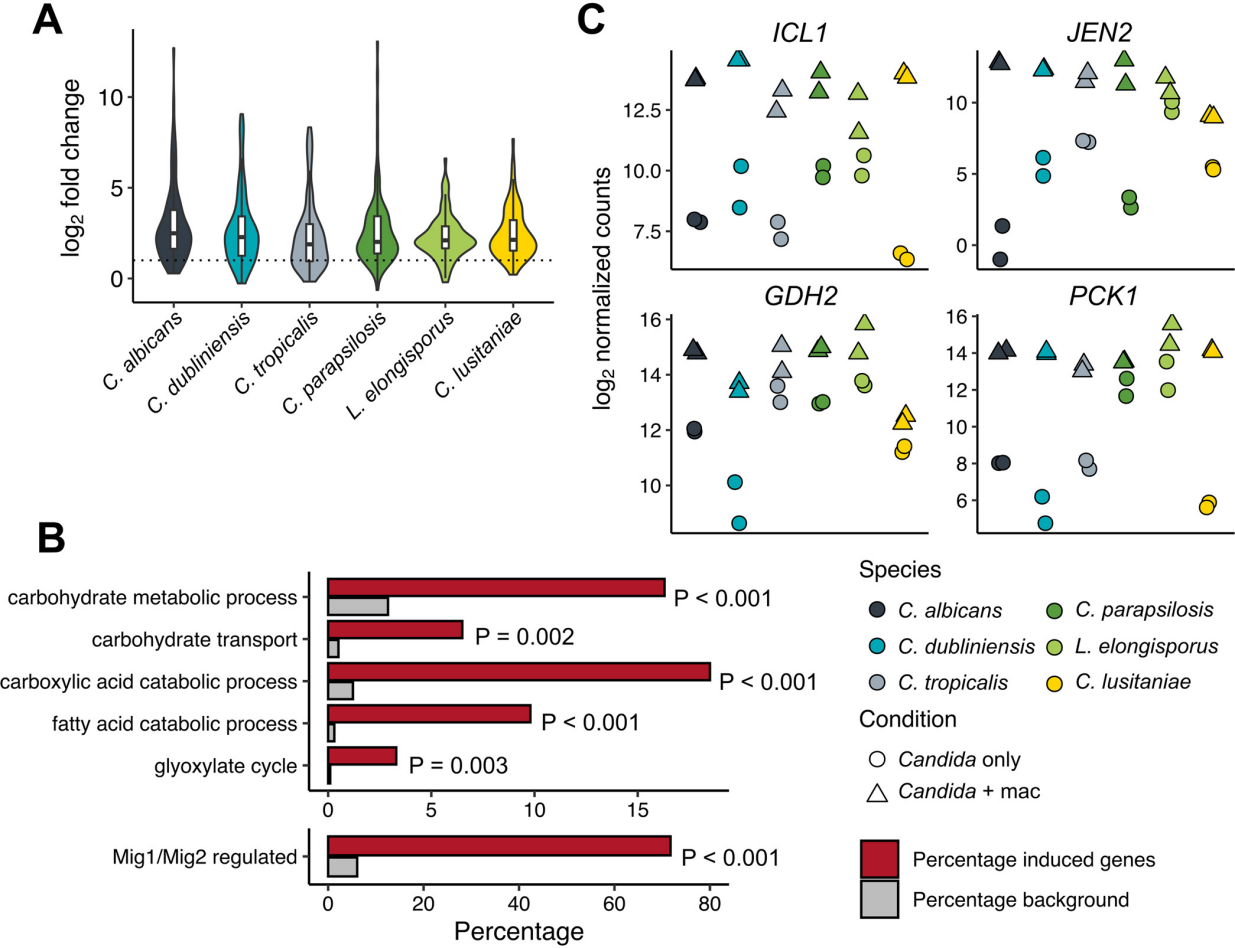
The core response of CUG clade species primarily consists of alternative carbon metabolism genes. (A) Violin plot of the log_2_ fold change (LFC) of the 92 genes showing significant induction (FDR = 0.1) across at least five species. Box plots showing median and quartiles are superimposed. The dashed line indicates an LFC of 1 (2-fold induction). (B) Selected enriched GO terms (top) and Mig1/Mig2 regulon inclusion (bottom) among core induced genes depicted in panel A. Analysis was performed using C. albicans orthologs, and the 4,376 conserved genes were the background gene set. FDR-adjusted *P* values for GO term enrichment are shown. The *P* value for Mig1/Mig2 regulon inclusion is from a hypergeometric test comparing core induced and total genes. (C) Examples of individual alternative carbon metabolism genes demonstrating conserved phagocytosis-dependent induction.

In order to investigate further the general inductive response, we identified genes significantly induced in at least five out of six species, revealing a conserved core set of 92 genes that overall showed similar degrees of induction across species (Fig. 2A; Table S1). These genes were enriched for gene ontology (GO) terms reflective of a broad metabolic response to carbon starvation, including lipid, carboxylic acid, and carbohydrate metabolism (Fig. 2B). This suggested a role for carbon catabolite repression (CCR) in mediating the expression changes we observed. CCR is mediated in S. cerevisiae by the Mig1 transcriptional repressor (37, 38) and in C. albicans by the partially redundant orthologs Mig1 and Mig2 (39). Of 259 Mig1/Mig2-repressed genes conserved across all species (6.1% of background), 66 were included in the 92 core induced genes (71.7%, *P* = 3.31 × 10^−64^, hypergeometric test), suggesting that derepression in low glucose is an important driver of the conserved response (Fig. 2B). Indeed, we examined the normalized read counts for four highly induced genes involved in the glyoxylate cycle (*ICL1*), amino acid catabolism (*GDH2*), dicarboxylic acid uptake (*JEN2*), and gluconeogenesis (*PCK1*) (Fig. 2C). To our surprise, despite the differences in fold induction between species, expression of each of these genes in phagocytosed cells converges to similar levels in all species. Conversely, only seven genes were repressed in at least five species, with functions relating to ribosome biogenesis and translation (Table S1) Therefore, both the PCA and differential expression approaches identified that the broad induction of metabolic genes previously reported in C. albicans (17, 18), as well as the non-CUG species Candida glabrata (24), is conserved across all species of the CUG clade, although the set of repressed genes appears to be less so.

In addition to nutritional limitation, phagocytosed microbes must also respond to oxidative and nitrosative stresses. *CAT1*, which has a crucial role in reactive oxygen species (ROS) detoxification (40), was included in the core induced gene set (Table S1; Fig. S2), as were the oxidoreductase *CIP1* and the thiol-specific peroxiredoxin *AHP2*, which have also been implicated in the oxidative stress response. In contrast, the nitric oxide dioxygenase *YHB1*, which is required for resistance to nitrosative stress (41), was significantly induced only in C. albicans, C. tropicalis, and *L. elongisporus* (Fig. S2).

10.1128/mbio.03317-21.2 FIG S2 Expression of stress response genes *CAT1* and *YHB1*. Log-transformed fragment counts for *CAT1* (*C1_06810W* in C. albicans) and *YHB1* (*CR_07790C*) ortholog groups are shown. Download FIG S2, TIF file, 0.3 MB. Copyright © 2021 Pountain et al. 2021 Pountain et al. https://creativecommons.org/licenses/by/4.0/ This content is distributed under the terms of the Creative Commons Attribution 4.0 International license.

### Interspecies differences in gene induction upon phagocytosis are often driven by variation in prephagocytosis expression levels.

**FIG 3:**
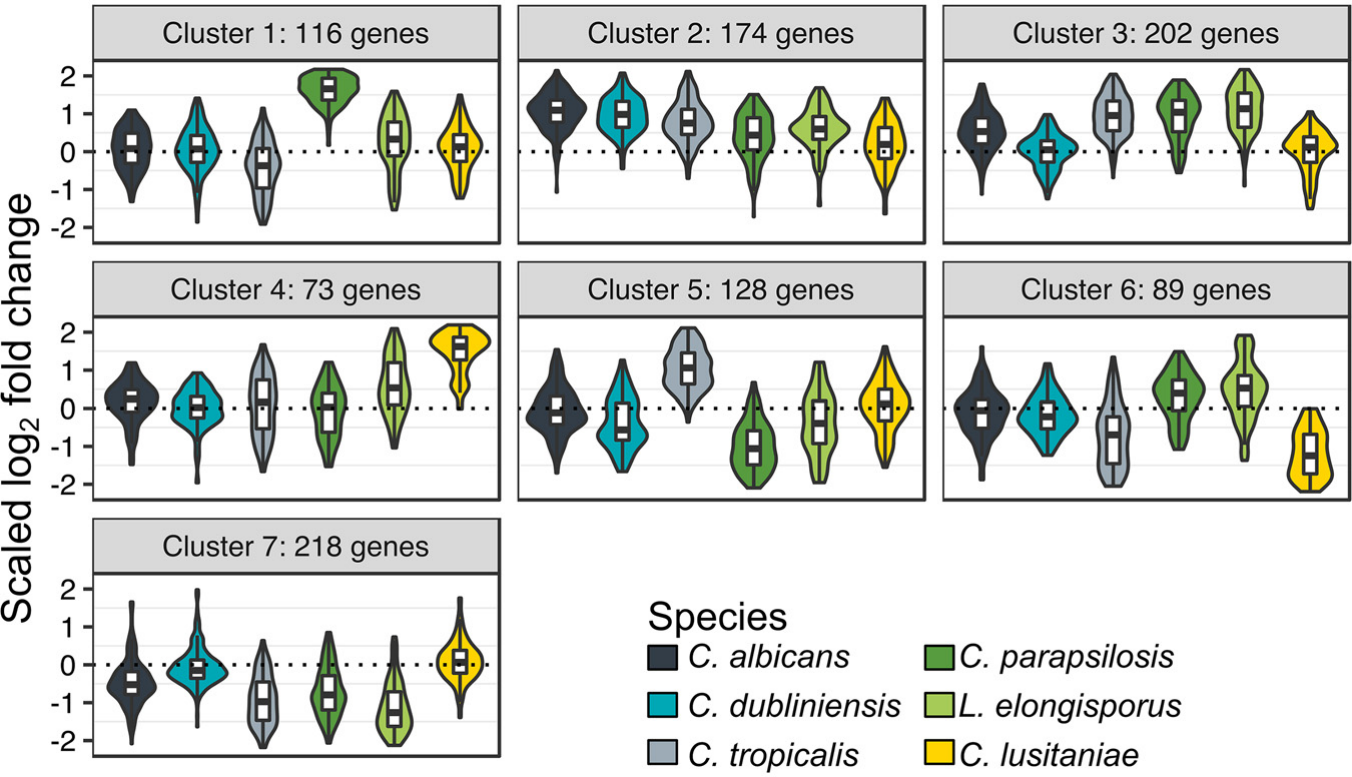
Cluster analysis reveals species-specific differences in phagocytosis response. *k-*medoids clustering of the 1,000 genes showing the most variance in log_2_ fold change (LFC) across species. LFCs are scaled by division by the root mean square. Scaled LFCs for each cluster are shown as violin plots, with box plots showing median and quartiles superimposed. Plots are color coded by species according to the legend.

While there was substantial overlap in the response to phagocytosis, we wished to identify genes that exhibited variable responses between species. Therefore, of 4,259 genes that averaged at least 10 normalized read counts in every species, we identified the 1,000 genes with the highest variance in log_2_ fold change (LFC) across species. These genes (23.5% of the 4,259 conserved genes) accounted for 63.5% of the total variance, and so this captures most of the species-specific differences in phagocytosis response. We performed *k*-medoids clustering, which is more robust to outliers than the related *k*-means method, to assign these genes to seven clusters (Fig. 3; Table 2). Clusters 1 to 5 all consisted of genes induced in at least some species. Cluster 1 consisted of genes induced most highly in C. parapsilosis, with this cluster showing no functional enrichments (although 14 out of 116 genes have a putative transmembrane transport role); cluster 4 contains genes induced most highly in C. lusitaniae, which are enriched for functions in hexose metabolism and transport. Genes in clusters 2 and 3 both were broadly induced, with cluster 2 showing the strongest induction in C. albicans (enriched for a number of alternative carbon metabolism pathways), and cluster 3 showing particularly strong induction in C. tropicalis, C. parapsilosis, and *L. elongisporus* (enriched for organic acid and amino acid metabolism and peroxisome organization). In contrast, cluster 7 showed broad repression of ribosome biogenesis and noncoding RNA processing genes in all species except C. dubliniensis and C. lusitaniae. This implies that the repressive response reported in C. albicans is unevenly conserved, although in other species, such as *L. elongisporus*, it may be even stronger than in C. albicans. Downregulation of cluster 7 pathways required for cell growth also suggests that most species face stress-induced growth arrest upon phagocytosis.

**TABLE 2:** Characteristics of clusters partitioned by *k*-medoids clustering^*a*^

Cluster	No. of genes	Characteristic	Enriched functional groups
1	116	Up in C. parapsilosis	NA
2	174	Up generally, but more in C. albicans/C. dubliniensis	Organic acid metabolism, lipid metabolism, amino acid metabolism
3	202	Up in C. albicans, C. tropicalis, C. parapsilosis, *L. elongisporus*	Organic acid metabolism, amino acid metabolism, peroxisome organization
4	73	Up in C. lusitaniae	Hexose metabolism and transport
5	128	Up in C. tropicalis, down in C. parapsilosis	Sulfate assimilation, cytoplasmic translation
6	89	Down in C. tropicalis and C. lusitaniae	Septation initiation signaling
7	218	Down in C. albicans, C. tropicalis, C. parapsilosis, *L. elongisporus*	Ribosome biogenesis, ncRNA processing, aspartate metabolism

a For each cluster as visualized in Fig. 3, the number of genes, characteristic pattern, and functional groupings based on GO term analysis are shown. Full cluster information is found in Table S2. NA, not available; ncRNA, noncoding RNA.

10.1128/mbio.03317-21.8 TABLE S2 Clusters of genes as partitioned by *k*-medoids clustering analysis and gene ontology (GO) term enrichment analysis of individual gene clusters. Cluster analysis and GO term enrichment analysis were performed as described in Materials and Methods, with individual clusters described in Fig. 3 and Table 2. For each cluster, the gene ID, baseMean (the mean expression across all samples of that species), the log_2_ fold change (LFC), and the multiple comparisons-adjusted *P* value (padj) are shown for each species, along with the C. albicans gene description and name. In separate tabs, results of GO term analysis of each cluster are shown. Note that no enriched GO terms were found for cluster 1. Download Table S2, XLSX file, 0.4 MB. Copyright © 2021 Pountain et al. 2021 Pountain et al. https://creativecommons.org/licenses/by/4.0/ This content is distributed under the terms of the Creative Commons Attribution 4.0 International license.

**FIG 4:**
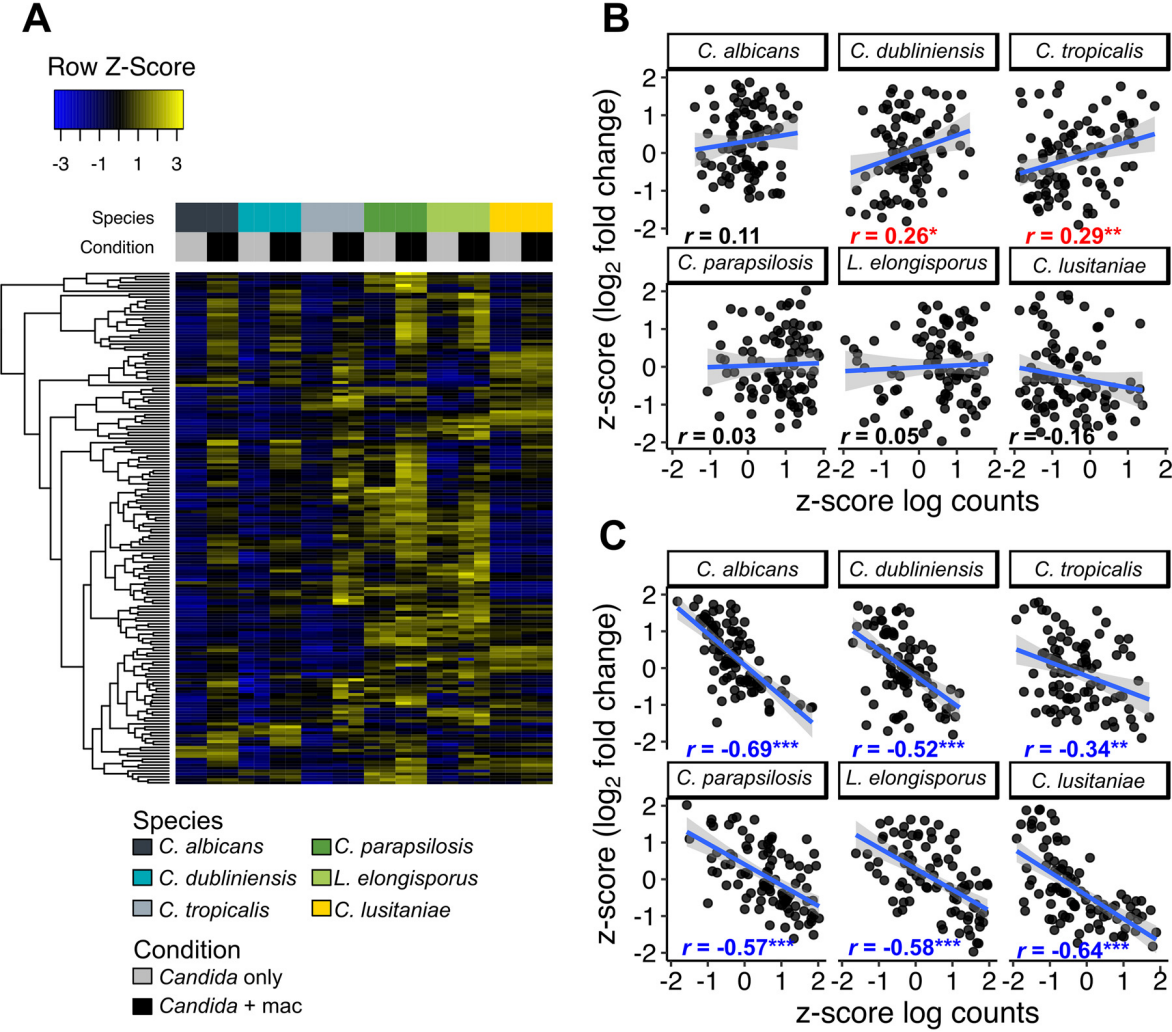
Variably induced genes show convergence in expression levels upon phagocytosis. (A) Heatmap of mean-centered, log-transformed fragment counts of genes in cluster 2 (Fig. 3). Hierarchical clustering of genes was performed using Euclidean distances. (B and C) For all 92 core response genes, log_2_ fold change and mean log-transformed gene counts for *Candida* with macrophage samples (B) or *Candida*-only samples (C) were converted into z-scores. Plots are shown for each species individually with a linear regression line fitted (in blue, with gray-shaded areas representing 95% confidence intervals). Pearson correlation coefficients are shown for each species, and asterisks mark a significant relationship between z-scores (linear model, *, *P* < 0.05; **, *P* < 0.01; ***, *P* < 0.001). Blue text indicates a significant inverse relationship, and red indicates a significant positive relationship.

Genes in cluster 2 show the strongest response in C. albicans, and most of the 92 core genes are in this cluster. However, the greater induction did not, in general, result in higher expression relative to other species; instead, the high induction ratios in C. albicans derived from unusually low expression under the control (nonphagocytosed) condition (Fig. 4A), as we had observed with some individual genes (Fig. 2C). In other words, the LFC values were primarily driven not by expression levels in phagocytosed cells but by the expression under the control condition, which varied significantly between species. In general, we observed good reproducibility across replicates and at least modest expression in nonphagocytosed cells (Fig. 2C). For a few phagocytosis-induced genes, variation in LFC could be exaggerated due to the inherent noisiness of measurement of very low baseline expression levels (e.g., *JEN2*).

To determine whether this trend was also observed in the core inductive response, we compared z-scores for gene expression in the presence or absence of macrophages with z-scores for LFC across all species and all 92 genes in this set (Fig. 4B and C). The correlation between LFC and expression was surprisingly poor for the phagocytosed samples (Pearson’s *r* ranging from −0.15 to 0.29 [Fig. 4B]). In contrast, all species showed a significant negative relationship between LFC and expression in *Candida*-only samples (Pearson’s *r*, −0.34 to −0.69 [Fig. 4C]). To assess whether these correlations were a general feature of the data, we calculated *r* for each species across 10,000 randomly selected sets of 92 genes and found that for every species except C. dubliniensis, there was a stronger negative correlation between LFC and *Candida*-only counts in the core induced genes than 99% of randomly selected gene sets (Fig. S3). Paradoxically, therefore, higher postphagocytosis induction in core induced genes in C. albicans does not result from higher expression in the macrophage but from lower expression under the control condition, which we propose to be a product of tighter glucose repression in this species (see Discussion).

10.1128/mbio.03317-21.3 FIG S3 Correlations of fold change and transcript abundance for iteratively randomly selected subpopulations of genes. For all conserved genes, z-scores were calculated for mean log-transformed fragment counts and log_2_ fold changes for *Candida* in the presence (A) or absence (B) of macrophages. From 4,376 conserved genes, 92 genes (the size of the core induced gene set) were randomly selected, and Pearson’s *r* correlation coefficients were calculated for each species. This process was iterated 10,000 times to generate a distribution of expected *r* values based on the whole population. These distributions are shown, with the black dotted line indicating an *r* value of zero. The red dashed lines indicate *r* values for the core induced genes. While there is variation between species, in general *r* values for *Candida*-macrophage samples skew positive and *Candida-*only samples skew negative. With the exception of C. dubliniensis, *r* values for the core induced genes in *Candida-*macrophage samples are less positive than the general population, whereas *r* values for *Candida*-only samples are more negative than 99% of the general population. This indicates that core induced genes exhibit more convergence in gene expression upon phagocytosis than the general population. Download FIG S3, TIF file, 1.1 MB. Copyright © 2021 Pountain et al. 2021 Pountain et al. https://creativecommons.org/licenses/by/4.0/ This content is distributed under the terms of the Creative Commons Attribution 4.0 International license.

### C. albicans filamentation and biofilm formation genes are expressed more highly inside macrophages than their orthologs in other CUG clade species.

**FIG 5:**
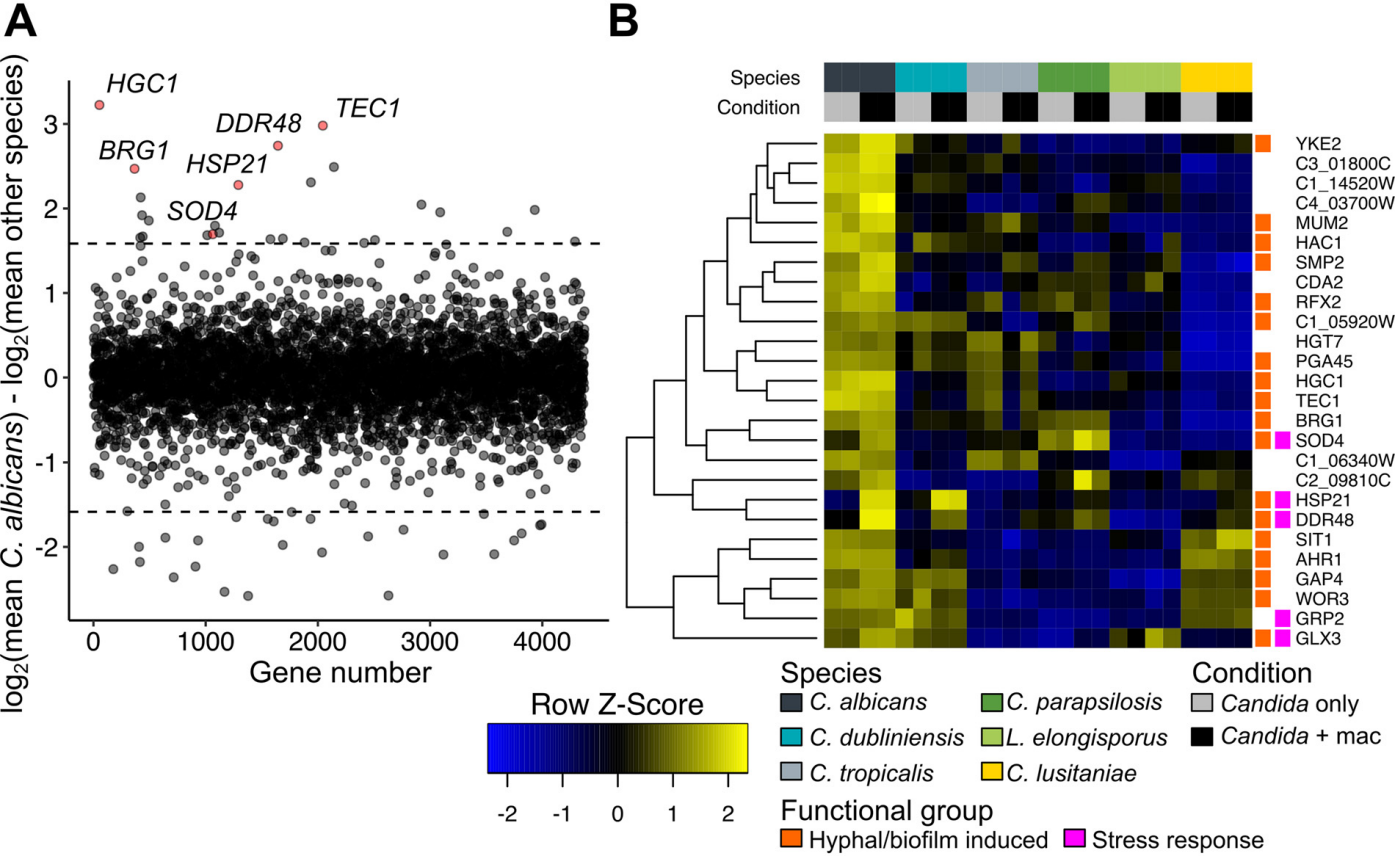
Identification of genes more highly expressed in C. albicans than other species. (A) The mean macrophage log-transformed fragment counts of all non-*albicans Candida* species were subtracted from the mean macrophage log-transformed fragment counts for C. albicans. Dotted lines indicate a 3-fold difference in expression (log_2_ difference of 1.58) in C. albicans from the mean for other species. Selected genes of interest are highlighted in red. (B) Heatmap of mean-centered, log_2_-transformed read counts of genes that are at least 3-fold more highly expressed in C. albicans macrophage samples compared to the mean for other species. Hierarchical clustering of genes was performed using Euclidean distances. Underlying data and equivalent analyses for C. parapsilosis and C. tropicalis are found in Table S3. Genes previously associated with either hyphal morphogenesis or biofilm formation, as well as stress responses, are indicated.

The curious observation that many genes converged on similar expression levels, and thus their strong induction in response to phagocytosis in some species was a reflection of lower expression under control conditions, led us to consider whether absolute expression may, in some cases, be a better indicator of biological functions required for virulence in this model. To do this, we determined the difference of log-transformed expression in C. albicans within a macrophage from the mean expression across all other species (Table S3). Only 1.2% of C. albicans genes differed from orthologs in related species by 3-fold in expression (equivalent to a 1.58 difference in log_2_ expression), 26 of which showed increased expression (Fig. 5A). This set was especially enriched for genes implicated in biofilm formation and hyphal growth, as 14 were annotated as biofilm or hypha induced or had related phenotypes when mutated (Table S3, based on *Candida* Genome Database annotation [42]). This set included several key regulators such as *TEC1*, *BRG1*, *AHR1*, *WOR3*, and *HAC1* and the hypha-specific cyclin *HGC1*. Four other genes, *MUM2*, *SMP2*, *RFX2*, and *YKE2*, are regulated by the global transcriptional repressors Tup1 and Nrg1, which themselves have strong morphogenetic phenotypes (43, 44). Thus, 18 of the 26 genes identified that are more highly expressed in C. albicans than in other species are positively linked to morphogenesis (two others, *HGT7* and *C1_14520W*, are repressed in biofilms). This observation is consistent with the known importance of hyphal differentiation and biofilm development in C. albicans compared to other *Candida* species (33). We performed similar analyses for the other two common pathogens, C. tropicalis and C. parapsilosis, and while no enriched groups were found in highly expressed genes in C. tropicalis, C. parapsilosis showed especially strong expression of fatty acid catabolism genes (*FOX2*, *POX1* to -*3*, *PXP2*) (Table S3). The homologs of catalase (*CAT1*) and a superoxide dismutase (*SOD4*) are also in this group, indicating a robust ability of C. parapsilosis to detoxify ROS, as demonstrated previously (33).

10.1128/mbio.03317-21.9 TABLE S3 Comparison of mean expression inside macrophages for C. albicans, C. parapsilosis, and C. tropicalis to the mean of other species. Underlying data for Fig. 5. For each species, the mean log-transformed fragment counts in macrophages for that species and for all other species are shown, along with the difference between them. This is converted into an equivalent fold change derived from the transformed data (regularized log-transformed data as generated by the rlog function in DESeq2 are subject to log_2_ transformation, but with additional normalization for library size and variance stabilization for rows with small counts). For C. parapsilosis and C. tropicalis, abundance ratios and heatmaps of atypically highly expressed genes are shown as described for C. albicans in Fig. 5. Download Table S3, XLSX file, 1.3 MB. Copyright © 2021 Pountain et al. 2021 Pountain et al. https://creativecommons.org/licenses/by/4.0/ This content is distributed under the terms of the Creative Commons Attribution 4.0 International license.

**FIG 6:**
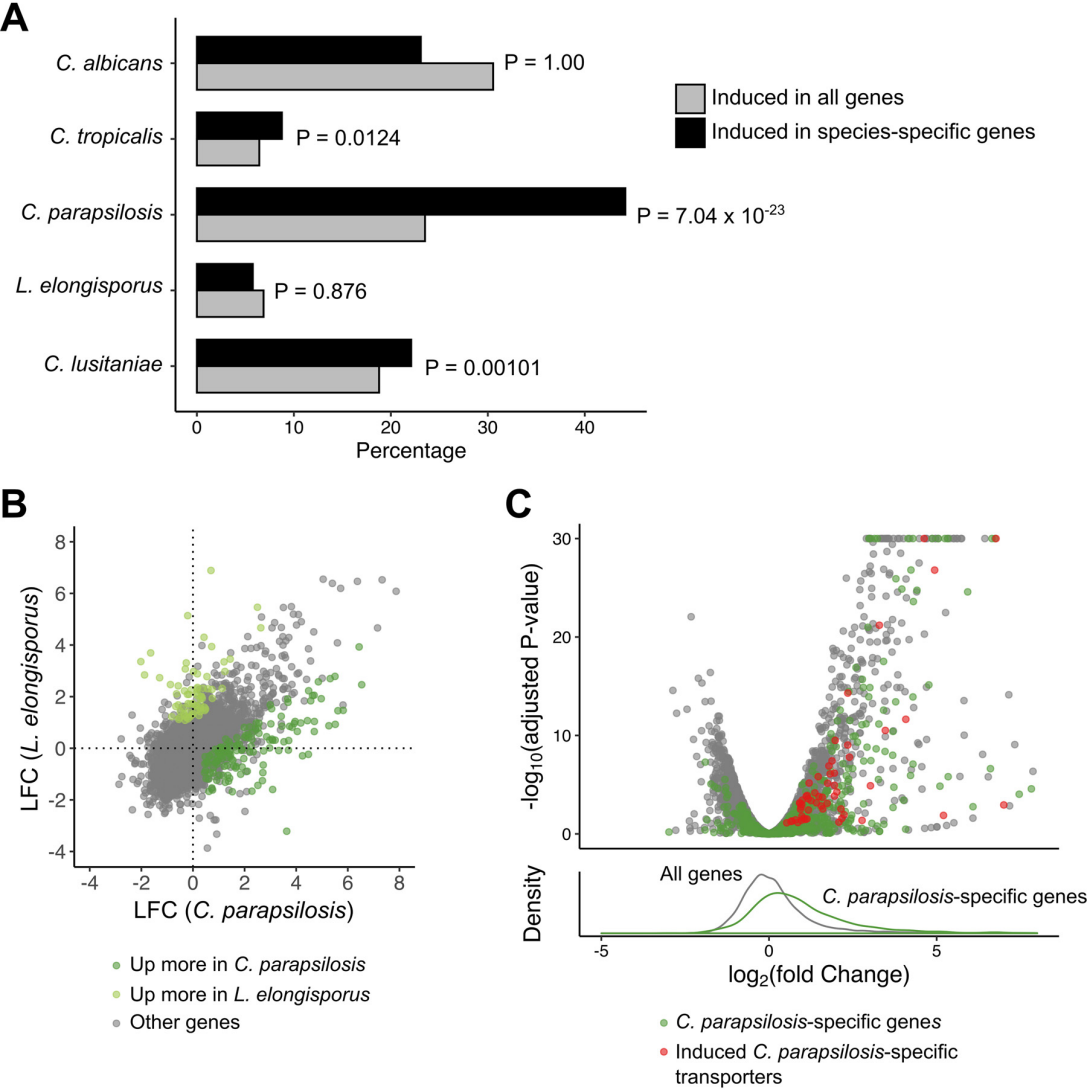
C. parapsilosis has an enrichment of phagocytosis induction in species-specific genes and enhanced transporter induction compared to related *L. elongisporus*. (A) For each species, the fraction of unique genes (found only in that species) induced is compared to the fraction of all genes induced in that species. *P* values are calculated using hypergeometric tests. C. dubliniensis was excluded, as it shares nearly all genes with C. albicans. (B) Comparison of log_2_ fold change (LFC) between C. parapsilosis and *L. elongisporus* in response to phagocytosis. (C) Volcano plot of genes in the C. parapsilosis response to phagocytosis (upper panel). C. parapsilosis genes without an ortholog in *L. elongisporus* are in green (C. parapsilosis-specific induced putative transporter-encoding genes are in red). A histogram (lower panel) of the distribution of LFC for all C. parapsilosis genes (gray) versus C. parapsilosis-specific genes (green) demonstrates an enrichment for the species-specific genes among the induced genes.

Higher expression of several stress-related genes was also observed (Fig. 5B), and a few of these are both more highly expressed in C. albicans than in other species and show strong induction in response to phagocytosis (Fig. 5B), with three being induced at least 3-fold (*HSP21*, *DDR48*, and *SOD4*), each of which has been linked to virulence in C. albicans (45–47). Of these, *HSP21* showed the strongest induction (112-fold) (Fig. 6A). *HSP21* is poorly expressed in C. parapsilosis and not induced by phagocytosis (Fig. S4). As this species is relatively heat sensitive, we asked whether overexpression of *HSP21* in C. parapsilosis would be sufficient to improve heat tolerance, but it did not. When *CpHSP21* was expressed under the C. albicans promoter (in C. parapsilosis), it was induced in response to heat and phagocytosis, indicating a retained ability of this species to respond to these conditions (Fig. S4).

10.1128/mbio.03317-21.4 FIG S4 Expression of *HSP21* and thermotolerance are attenuated in C. parapsilosis. (A) Expression of *HSP21* orthologs in RNA-seq data across species in response to phagocytosis is shown as log-transformed, normalized fragment counts. (B) qRT-PCR expression analysis of *Candida* species in response to phagocytosis. Bars represent mean log-transformed fold changes of *Candida* with J774 macrophages versus *Candida* in mammalian culture medium alone. (C) qRT-PCR analysis of *Candida* species in response to heat stress. Bars represent mean log-transformed fold changes of *Candida* grown in YPD for 30 minutes at 42°C versus 30°C. (D) Growth of *Candida* species at different temperatures. Fivefold dilutions of *Candida* in water were spotted onto YNB-glucose agar and incubated for 48 h at the temperatures shown. (E) Growth of C. parapsilosis transformant lines at different temperatures. Performed as in panel D. (F) qRT-PCR analysis of *HSP21* expression in C. parapsilosis transformant lines. Bars represent mean log_2_-transformed fold changes compared to wild type at 30°C. (G) qRT-PCR analysis of *HSP21* expression in C. parapsilosis transformant lines. Bars represent mean log_2_-transformed fold changes compared to wild-type *Candida* in mammalian culture medium alone (-J774). All growth assays and qRT-PCR experiments were performed in three independent biological replicates. Error bars indicate standard deviation. *, *P* < 0.05; **, *P* < 0.01; ***, *P* < 0.001. *P* values were derived from Student’s *t* tests performed on δ*C_T_* values to determine significant induction (B and C) or δδ*C_T_* values to determine species-specific differences in induction. Download FIG S4, TIF file, 1.6 MB. Copyright © 2021 Pountain et al. 2021 Pountain et al. https://creativecommons.org/licenses/by/4.0/ This content is distributed under the terms of the Creative Commons Attribution 4.0 International license.

### C. parapsilosis demonstrates broader induction of nutrient transporter genes than its relative *L. elongisporus*.

To determine whether there were additional species-specific adaptations to the host environment, we took two approaches using LFC as our primary tool. We first investigated whether genes that were unique to individual species were induced in response to phagocytosis. For each species, we quantified the percentage of conserved and unique genes induced in response to phagocytosis, applying a hypergeometric test to determine whether species-specific genes were enriched (Fig. 6A). Interestingly, strong evidence of this was identified only in C. parapsilosis, with 44% of 421 genes unique to this species induced compared to only 24% of background. This suggests that these species-specific genes, which are more likely to be recently acquired during speciation, are particularly important adaptations to the host environment.

**FIG 7:**
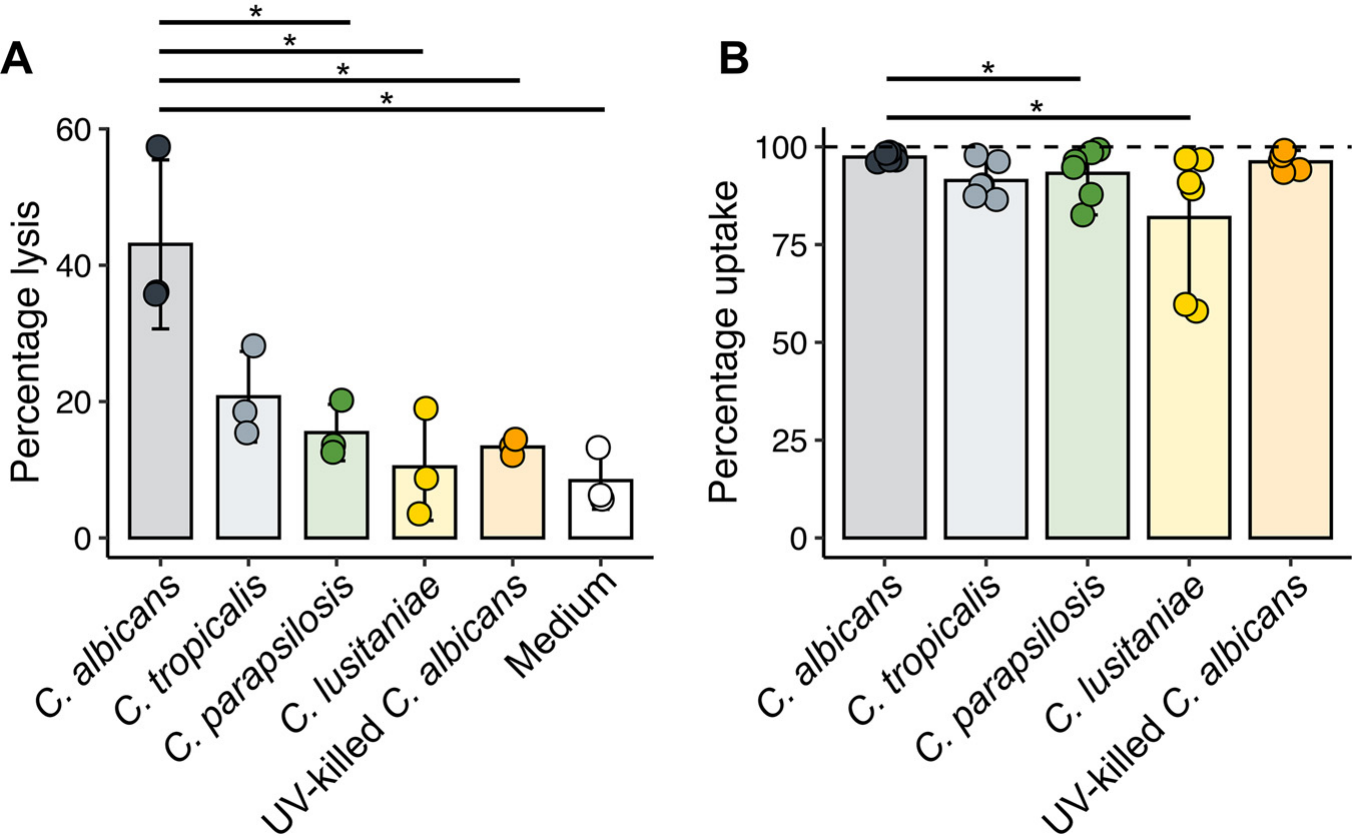
*Candida* species show variable macrophage lytic ability despite similar phagocytosis rates. (A) Percent lysis of macrophages by different *Candida* species. Macrophages were coincubated with *Candida* or medium only at a 3:1 MOI for 6 h. Lysis was measured by lactate dehydrogenase activity in the supernatant, with data shown as a percentage of activity of samples subjected to complete chemical lysis. *, *P* < 0.05, pairwise Student’s *t* test. Bars indicate the mean from three independent experiments, with error bars indicating the standard deviation. (B) Percentage of macrophages engulfing at least one *Candida* cell. Macrophages and *Candida* cells were coincubated at a 3:1 MOI for 1 h, before staining to identify internalized *Candida* cells as described in Materials and Methods. Representative images are shown in Fig. S5. *, *P* < 0.05, Wilcoxon signed-rank test. Bars indicate the mean from six independent experiments, with error bars indicating the range.

While C. parapsilosis shows reduced virulence in mouse models compared to C. albicans, it is nevertheless a frequent cause of candidemia, whereas its close relative *L. elongisporus* is very rarely isolated in patients (Fig. 1A). Among conserved genes, we saw overall a high degree of overlap both by PCA (Fig. 1B) and by comparing LFC estimates (Fig. 6B). However, we did identify 140 genes induced to a higher degree in C. parapsilosis (Table S1), including 26 transmembrane transporters that represented a significant enrichment (false-discovery-rate [FDR]-adjusted *P* value of 0.00023). These transporters included six putative amino acid and three oligopeptide transporters (notably C. parapsilosis orthologs of *CAN1*, *GAP2*, *OPT1*, *PTR2*, and *PTR22*). Peptides and amino acids have been proposed to be important nutrients in some host niches (48), and CUG clade species efficiently use amino acids as a carbon source (33). To broaden this analysis, we used the Candida Gene Order Browser (CGOB) (35) to identify 666 genes in C. parapsilosis without an annotated ortholog in *L. elongisporus*, of which 262 were induced in response to phagocytosis. Of the 93 C. parapsilosis-specific genes encoding putative transmembrane transporters, 56 were induced in response to phagocytosis (Fig. 7B), including 11 putative amino acid transporters and two putative oligopeptide transporters. Therefore, C. parapsilosis has dramatically expanded its repertoire of transmembrane transporters and these genes are much more likely than conserved genes to be induced under host-relevant conditions relative to *L. elongisporus*, consistent with an increased need for nutrient uptake *in vivo*.

10.1128/mbio.03317-21.5 FIG S5 Representative microscopy images of *Candida* uptake by macrophages. *Candida* was coincubated with macrophages at an MOI of 3 for 1 h before fixing and staining as described in Materials and Methods. Extracellular fungi were stained with Alexa Fluor 594-conjugated concanavalin A (red), before permeabilizing and staining all fungi with FITC-conjugated anti-C. albicans rabbit polyclonal antibody (green) and staining macrophage nuclei with NucBlue (blue). Quantification of all samples is represented in Fig. 7B. Download FIG S5, TIF file, 2.7 MB. Copyright © 2021 Pountain et al. 2021 Pountain et al. https://creativecommons.org/licenses/by/4.0/ This content is distributed under the terms of the Creative Commons Attribution 4.0 International license.

### Species-specific responses include many uncharacterized genes.

The changes in gene content exemplified by the expansion in transport functions in C. parapsilosis highlight one mechanism of species-specific adaptation. Changes in the regulation and expression of conserved genes are another. We again used LFC measures to determine the unique macrophage-induced regulon of the most significant pathogens C. parapsilosis, C. albicans, and C. tropicalis (Table 1). C. parapsilosis again had the broadest response, inducing 170 conserved genes. Seventeen of these genes encode predicted transcription factors, including the homologs of *WOR1*, *CZF1*, *CAP1*, *MRR1*, *UME6*, and *SEF2*, all of which have roles in host-pathogen interactions in C. albicans. None of these genes have been studied directly in C. parapsilosis. Surprisingly, 53 of the 170 genes (31%) lack an ortholog in S. cerevisiae, suggesting again that they are more recent adaptations to the host environment. The orthologs for 13 additional genes are themselves uncharacterized in yeast.

The 76 uniquely upregulated genes in C. albicans are enriched for genes predicted to play roles in autophagy, though these are mostly functions inferred from homology. Autophagy is another response to nutrient starvation (and cellular damage); this process contributes to the survival of C. glabrata in macrophages (49, 50), though its role in C. albicans is less clear (51). The majority of the upregulated genes are uncharacterized, and 23 lack a yeast ortholog (30%). Considering C. albicans, C. tropicalis, and C. dubliniensis as a group, the only significant functional annotation term for the 130 genes upregulated only in one or more of those species is “uncharacterized.” These gene sets are thus a potentially important source of pathogen-specific adaptations that should be studied further.

### Macrophages produce an early transcriptional response that is independent of fungal species and viability.

**FIG 8:**
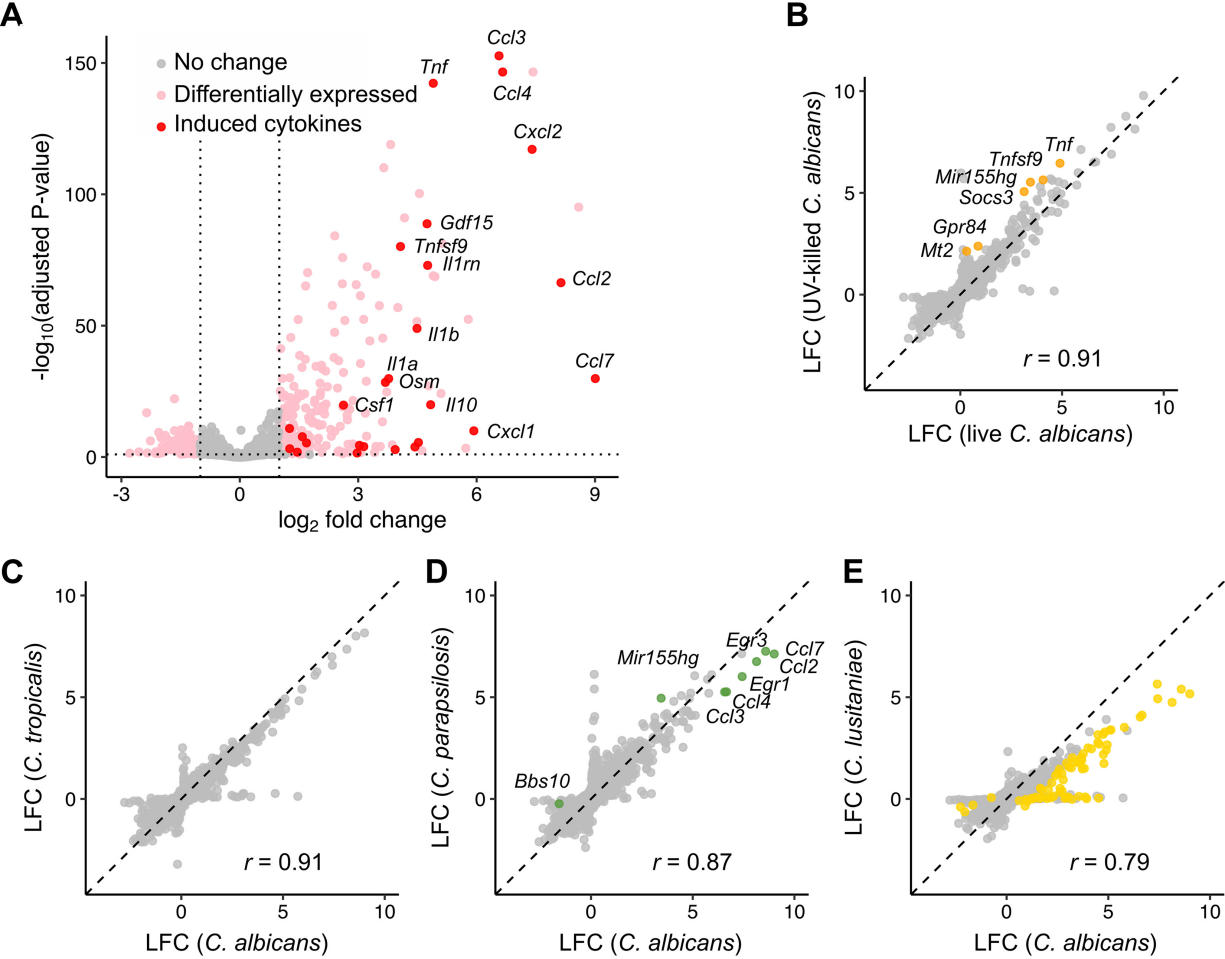
Different *Candida* species elicit similar proinflammatory responses in macrophages that are independent of fungal viability. (A) Volcano plot of expression changes in C. albicans-stimulated macrophages compared to unstimulated controls. Induced cytokines are colored red with several of the most strongly induced cytokines labeled. (B to E) Log_2_ fold change in macrophage gene expression in response to live C. albicans compared to UV-killed C. albicans (B), C. tropicalis (C), C. parapsilosis (D), and C. lusitaniae (E). Colored points indicate genes that are differentially expressed compared to the C. albicans-stimulated condition. Pearson correlation coefficients (*r*) for each comparison are shown.

The fungal response to phagocytosis contained both conserved and species-specific elements. We hypothesized that there would be a similar dichotomy in macrophage responses: most of these species elicit comparable levels of tumor necrosis factor alpha (TNF-α), for instance (52), while only C. albicans caused lysis of primary bone marrow-derived macrophages (BMDMs) (although C. tropicalis showed a trend toward higher lysis, *P* = 0.054 [Fig. 7A]). To assess these responses, we performed transcriptome sequencing (RNA-seq) analysis of macrophages from three mice (Table S4), stimulated at a 3:1 multiplicity of infection (MOI) for 1 h, after which nearly all macrophages had taken up at least one fungal cell (although more variable uptake was observed for C. lusitaniae [Fig. 7B]). Focusing first on the response to C. albicans, we identified 220 induced and 101 repressed genes (Fig. 8A). Using DAVID (53, 54) to identify functional groupings among induced genes, we found a strong enrichment (*P* = 8.5 × 10^−20^) of genes with “cytokine activity.” Genes encoding several proinflammatory cytokines were strongly induced, including interleukin-6 (IL-6), TNF-α, IL-1α, and IL-1β, as well as anti-inflammatory cytokines (IL-10, IL-1rn). DAVID analysis additionally revealed enriched signatures for chemotaxis of neutrophils (*P* = 1.9 × 10^−8^), lymphocytes (*P* = 2.1 × 10^−4^), monocytes (*P* = 5.9 × 10^−4^), and eosinophils (*P* = 6.0 × 10^−3^), suggesting that recruitment of other immune effectors is a prime outcome of macrophage stimulation. Previous transcriptomic data from murine bone marrow-derived macrophages exposed to C. albicans (18) showed similar induction of several chemokine genes (*Ccl3*, *Ccl4*, *Cxcl1*, *Cxcl2*, *Cxcl3*) as well as proinflammatory cytokines (*Il1a*, *Il1b*, *Tnf*, *Il6*). Among C. albicans-induced genes, 77% were also induced in classically M1-polarized macrophages (gamma interferon [IFN-γ]/lipopolysaccharide [LPS] [Fig. S6A]), although the response was weaker (median LFC 2.0, compared to 3.4 after IFN-γ/LPS stimulation).

**FIG 9:**
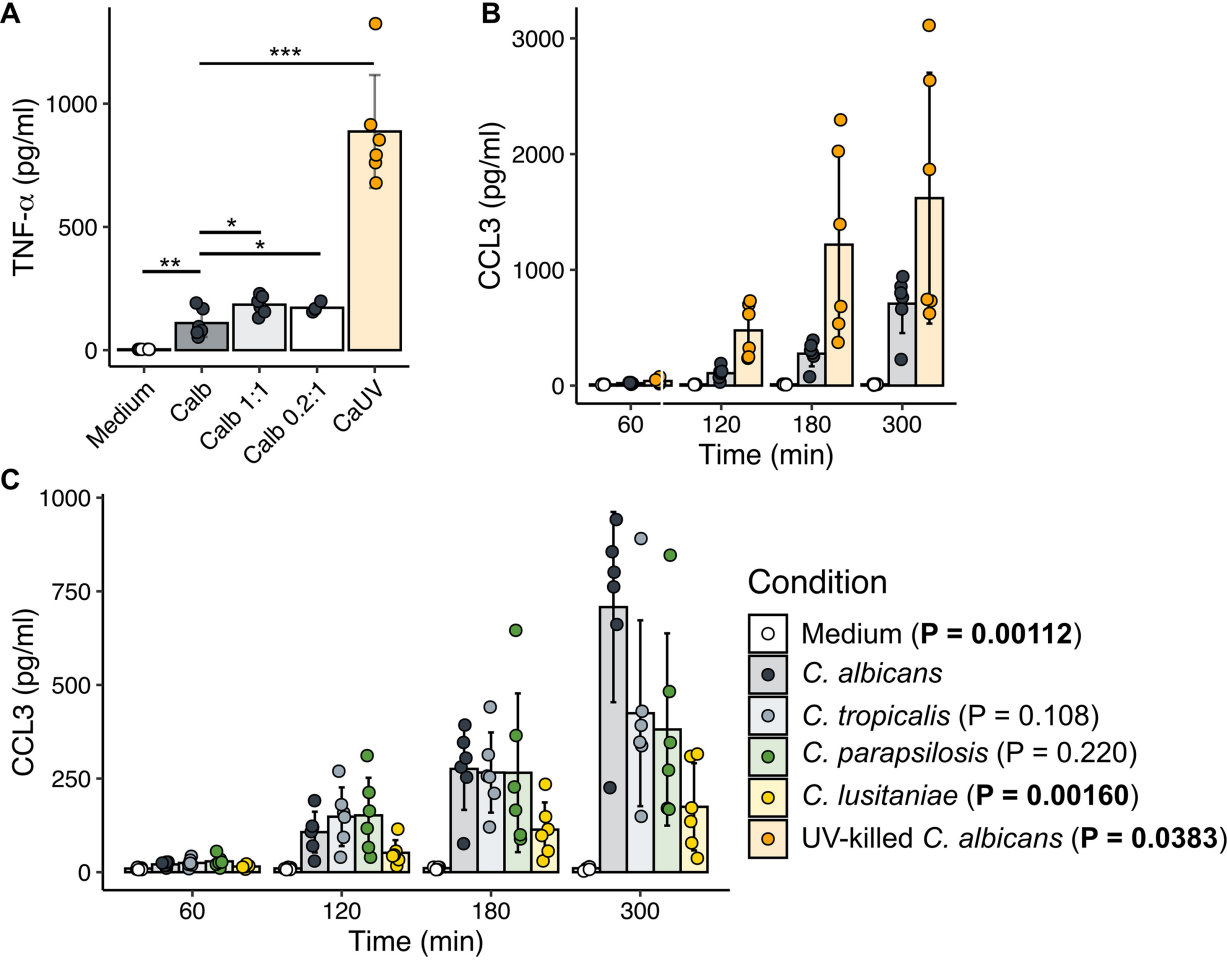
Cytokine secretion is influenced by *Candida* species and viability. (A) TNF-α secretion in response to live and UV-killed C. albicans as measured by ELISA of cell culture supernatants. Macrophages were stimulated for 5 h with medium, live C. albicans at various *Candida*-to-macrophage ratios (3:1, 1:1, and 0.2:1), or UV-killed C. albicans at a 3:1 ratio. ***, *P* < 0.001; **, *P* < 0.01; *, *P* < 0.05 (Student's *t* test versus C. albicans 3:1). Bar plots and error bars represent mean and standard deviation for six biological replicates. (B) CCL3 production over time as measured by ELISA of culture supernatants, comparing live and UV-killed C. albicans. (C) CCL3 production stimulated by different species. *P* values were determined by repeated-measures analysis of variance (ANOVA) compared to C. albicans-stimulated macrophages, and bar plots and error bars represent mean and standard deviation for five biological replicates.

10.1128/mbio.03317-21.6 FIG S6 Macrophage responses to various stimuli compared to C. albicans. (A) Comparison of macrophage responses to C. albicans and costimulation with IFN-γ and LPS. Log-transformed fold changes are plotted on each axis. Purple dots indicate genes induced in response to both stimuli, whereas blue and red dots represent changes specific to C. albicans and IFN-γ/LPS, respectively. These are defined as genes that are significantly induced by one stimulus but not the other, with further differential expression when comparing the two stimulated conditions directly. (B) TNF-α secretion in response to *Candida* spp. Macrophages were exposed to either medium alone or *Candida* fungi of different species, and TNF-α production was measured by ELISA after multiple incubation times. Bar plots show mean TNF-α concentration in the supernatant with error bars representing standard deviation for six biological replicates. *P* values were determined by repeated-measures ANOVA comparing conditions to C. albicans. Note that data for medium and C. albicans-treated macrophages are identical to those in Fig. 9A. (C) Normalized gene expression counts for macrophage *Ccl3* expression under each condition. (D) Determination of *Ccl3* gene induction by qRT-PCR. Fold was measured relative to unstimulated controls (left). Mean fold change is represented by dashes flanked by error bars representing standard deviation. *, *P* < 0.5; **, *P* < 0.01; ***, *P* < 0.001, Student’s *t* test on δδ*C_T_* values (effectively −log_2_ fold change estimates). As there was a high variability in fold change dependent on replicate, fold changes relative to the fold change in C. albicans are also shown (right). The dotted line represents no difference in fold change compared to C. albicans. (E) The effect of *Candida* cell dose on CCL3 production. CCL3 production was measured by ELISA. *P* values were determined by repeated-measures ANOVA comparing different *Candida* doses. Based on six biological replicates for medium and C. albicans-treated macrophages and five for C. lusitaniae-treated macrophages. Download FIG S6, TIF file, 1.7 MB. Copyright © 2021 Pountain et al. 2021 Pountain et al. https://creativecommons.org/licenses/by/4.0/ This content is distributed under the terms of the Creative Commons Attribution 4.0 International license.

10.1128/mbio.03317-21.10 TABLE S4 Pairwise comparisons of macrophage RNA-seq data. Pairwise differential expression analysis was performed as described in Materials and Methods comparing conditions to either unstimulated macrophages (medium-only) or C. albicans-stimulated macrophages. Download Table S4, XLSX file, 17.3 MB. Copyright © 2021 Pountain et al. 2021 Pountain et al. https://creativecommons.org/licenses/by/4.0/ This content is distributed under the terms of the Creative Commons Attribution 4.0 International license.

In order to determine how much this response was due to active processes within the fungus, we compared macrophage responses to live and UV-killed C. albicans. We observed a strong correlation in LFC estimates (*r *=* *0.92 [Fig. 8B]), although six genes were more highly expressed in UV-killed C. albicans-stimulated macrophages, including the proinflammatory cytokine gene *Tnf.* Gene set enrichment analysis (GSEA [55, 56]) identified a modestly more inflammatory state in response to UV-killed C. albicans relative to live cells, driven by subtle changes in a number of cytokine genes including *Lif*, *Il1a*, *Il1b*, *Il6*, *Il10*, *Cxcl10*, *Ccl2*, and *Ccl7.* Therefore, the response to C. albicans is largely independent of fungal viability, although UV killing of the fungus leads to a slightly stronger inflammatory response.

We then incorporated responses to other species into the analysis. C. parapsilosis and C. tropicalis induced quantitatively similar responses, while transcriptional changes in response to C. lusitaniae were broadly weaker (Fig. 8C to E). Of the 220 genes with at least 2-fold induction in response to C. albicans, the median fold changes were similar between C. albicans, C. parapsilosis, and C. tropicalis (3.7-fold, 3.6-fold, and 2.8-fold, respectively) but weaker to C. lusitaniae (1.7-fold). No macrophage genes differed significantly between C. albicans and C. tropicalis. Only six genes differed in response to C. parapsilosis, four of which were chemokine genes (*Ccl2*, *Ccl3*, *Ccl4*, *Ccl7*) with the other two being transcriptional regulators, early growth response genes *Egr1* and *Egr3*. The response to C. lusitaniae, in contrast, showed 68 genes with lower expression than for C. albicans, including proinflammatory cytokines and chemokines (*Il1a*, *Il1b*, *Ccl2*, *Ccl3*, *Ccl4*, *Ccl7*, *Cxcl2*).

Finally, we determined whether these differences reflected changes at the protein level. After 5 h, TNF-α secretion was 8-fold higher in response to UV-killed than live C. albicans (Fig. 9A), while no significant differences were seen in live C. albicans compared to other species (Fig. S6B). This is unlikely to be due to the lytic effects of live C. albicans alone as dropping the MOI from 3 down to 0.2 did not increase TNF-α to response levels to UV-killed fungi. We then examined whether species-specific differences in *Ccl3* expression were reflected in CCL3 secretion over time. In contrast to transcript levels (Fig. S6C and D), decreased secretion of CCL3 in response to C. parapsilosis was not observed, but C. lusitaniae produced 4.1-fold less than C. albicans after 5 h (Fig. 9C). As differences in cell size could contribute to quantitative differences in receptor signaling, we determined the effect of reducing the MOI of C. albicans to 1, and of raising the C. lusitaniae MOI to 10, but this had no significant effect on CCL3 secretion (Fig. S6E). Lastly, in contrast to transcript abundance data, CCL3 secretion after 5 h was significantly increased by UV killing of C. albicans (Fig. 9B). As IL-1β secretion is dependent on posttranslational processing (57), and hence transcriptional changes may not be fully reflective of active protein abundance, we attempted to measure IL-1β by enzyme-linked immunosorbent assay (ELISA) of protein supernatants, but it was undetectable under all conditions up to 5 h (data not shown), suggesting that secretion over this time period is not significant.

## DISCUSSION

Several *Candida* species within the CUG clade cause human infections with various frequency, while others are rarely or never pathogenic. Most research has been focused on C. albicans, and as a result, a broad understanding of how different species interact with immune cells is lacking. Here, we compared the responses of six different *Candida* species to phagocytosis using a comparative transcriptomics approach. Our species selection captured a diversity of clinical prevalence and experimental virulence phenotypes *in vivo* (Fig. 1A) to extend the analysis to less- and nonpathogenic species, in order to determine whether differences in transcriptional responses upon initial contact with innate immune cells offer insight into these relevant host-pathogen interactions.

These species behave very differently when phagocytosed by macrophages (Fig. 1A). C. albicans rapidly and almost universally germinates, which is seen less frequently with C. dubliniensis and C. tropicalis, while all three species result in widespread macrophage death (33). Early in the interaction, C. albicans induces death in macrophages via pyroptosis (30, 31); later, macrophage death is driven by glucose starvation (18, 58). The mechanism(s) of macrophage killing by the other species is not clear. Candida guilliermondii, C. lusitaniae, and *L. elongisporus* do not form hyphae or damage macrophages (33). We sought here to determine whether these disparate outcomes were the result of unique transcriptional programs in either the macrophage or the fungal cells that would suggest underlying mechanisms. To this effect, we chose a single time point, a snapshot in essence, of the interaction at a point prior to which the physiological impacts (hyphal growth and macrophage death) were not yet apparent. We reasoned that this would identify transcriptional changes that may be the cause of the later effects rather than the effect of them, though this does lose some temporal dynamics of the interactions.

In C. albicans, the transcriptional program has been well described and is dominated by alternative carbon metabolism pathways, a response also seen in C. parapsilosis (59) and the non-CUG species C. glabrata (24). In contrast, a much narrower, weaker response to phagocytosis was observed in the model yeast S. cerevisiae (19), suggesting that robust induction of alternative carbon metabolism in response to phagocytosis is an adaptation of pathogenic yeasts. We show here for the first time that this robust alterative carbon metabolism response extends to nonvirulent species. Thus, while alternative carbon metabolism pathways are required for full virulence in both C. albicans and C. glabrata (19–23, 60), we show that this response is not sufficient for a species to be virulent. Adaptation to alternative carbon sources in C. albicans is linked to other virulence properties such as stress response and cell wall architecture (21, 61), and so its pathogenic importance may be indirect. This has parallels with previous work showing that loss of the nicotinic acid synthesis pathway, believed to be a host adaptation in C. glabrata, was also lost in related nonpathogenic *Nakaseomyces* species (62); thus, these conserved evolutionary changes are not necessarily directly linked to adaptation to the host.

We noted that within this core response, there is variation in the degree of phagocytosis-mediated induction between species, but that this often results in convergence to a similar level of expression within the macrophage. We believe that this arises from different degrees of Mig1- and Mig2-dependent CCR, with the phagolysosome representing the fully derepressed state. In C. albicans 0.01% glucose is sufficient to induce strong repression of glyoxylate cycle, gluconeogenesis, and fatty acid oxidation genes (63); this is well below the 0.45% found in the Iscove’s modified Dulbecco’s medium (IMDM) used for these experiments. CCR has not been investigated in other CUG clade species, but the threshold for glucose-mediated repression may differ from C. albicans.

We took two approaches to identify species-specific transcriptional responses. First, we focused on differences in expression ratios between species, with GO term analysis to identify processes upregulated in individual species. Somewhat surprisingly, C. parapsilosis was the only species in which we observed induction of a unique set of functionally related genes, enriched for genes encoding predicted metabolite transporters, particularly of amino acids and peptides. C. parapsilosis has a considerably expanded repertoire of nutrient transporters relative to the closely related *L. elongisporus* (13), and we found many of these that lacked a direct ortholog in *L. elongisporus* were induced upon phagocytosis. A quite high proportion (39%) of the C. parapsilosis*-*specific genes are induced upon phagocytosis, suggesting that these are recent adaptations to nutrient-poor host environments, such as the skin, which C. parapsilosis frequently colonizes (64), while, *L. elongisporus* has been found in a number of nutritionally rich environmental niches (65). It has been proposed based on the low level of heterozygosity that C. parapsilosis went through a population bottleneck relatively recently, which may have allowed it to regain virulence traits that had been lost (66). Expansion of inducible metabolite transport capacity in C. parapsilosis may allow greater flexibility compared to nonpathogenic species in nutritionally challenging infection environments.

C. parapsilosis also has the highest number of genes induced only in that species. Nearly all of these are uncharacterized, but 10% are homologs of C. albicans transcriptional regulators, many of which regulate pathogenic function. Curiously, there is limited overlap between these genes and a similar analysis of transcriptional regulators upregulated after contact with human THP-1 monocyte-like cells (67), which may arise from distinct responses to these cells relative to the primary mouse BMDMs we used. This group demonstrated that several of the transcriptional regulators they showed to be induced upon macrophage contact had host-relevant phenotypes, which suggests that the transcriptomics approach is effective at identifying candidate virulence factors. C. parapsilosis thus illustrates two mechanisms of adaptation, changes in gene content and changes in gene regulation compared to a related species. Evidence that the biofilm regulatory network has evolved between C. albicans and C. parapsilosis reinforces the need for species-specific studies (68).

Our second approach stemmed from the observation that there was a much stronger correlation between fold induction and expression under control conditions than there was to expression under the phagocytosed condition. We reasoned that absolute expression may also be informative. We therefore identified genes whose expression was significantly higher in one species than the others, irrespective of fold induction. Genes whose expression was markedly higher in C. albicans were dramatically enriched for those associated with hyphal or biofilm growth, consistent with the known role of hyphal morphogenesis as a major distinguishing factor in the lethality of this species in experimental models compared to others (C. tropicalis is also lethal in a mouse model [9, 10] and forms true hyphae but does so less readily [33] than C. albicans). Overexpressed gene sets specific to C. tropicalis did not show GO enrichment for specific functions, while that for C. parapsilosis included fatty acid metabolic functions and stress responses, consistent with the emphasis on nutrient acquisition in this species. Taken together, absolute expression and fold change are both valid and valuable tools for visualizing gene expression patterns under experimental conditions.

When we assayed macrophage responses to a subset of these species, we expected a similar combination of conserved and species-specific responses, given the differing outcomes. Similar to previous reports, we found that macrophages produce a strong proinflammatory cytokine response to C. albicans (18, 29). Yet, the macrophage expression program was largely independent of fungal species or, indeed, even viability. One exception to this was TNF-α, which is induced modestly at the transcript level and significantly more at the protein level in response to killed cells relative to live C. albicans. This is likely due to effects of fungal killing on the degree of exposure of immunostimulatory β-glucan, which in intact cell walls is masked by a mannan layer. Although UV killing (used here) is less disruptive than heat killing (69), it can still lead to increased β-glucan (70). This discrepancy between gene and protein expression is seen with other cytokines, such as IL-1β, and so this analysis should be extended to additional cytokine profiling. Between species, the only notable differences in macrophage gene expression came from C. lusitaniae, which produced a qualitatively similar but quantitatively weaker response. This may be due to the smaller cell size of these haploid cells (though increasing the MOI did not enhance the cytokine response) or due to differences in cell wall composition, which have been previously reported (71–73).

In a recent publication, Cuomo, Rao, and colleagues devised a method to separate macrophages that had taken up C. albicans cells from those in the same culture that had not, finding the transcriptional profiles of the two populations to be nearly identical (29). Our data with multiple species are consistent with this and suggest a model in which the first macrophages to contact a *Candida* cell initiate proinflammatory cytokine release that then drives expression programs in the rest of the population via paracrine signaling. Given the homogeneity of the responses, then, how are the outcomes so different? C. albicans-induced macrophage death can be divided into an early phase characterized by pyroptosis (30, 31) and a later phase drive by glucose starvation (18). There are perhaps other mechanisms that contribute to macrophage lethality as well (32, 58). The lethal species (C. albicans, C. tropicalis, C. dubliniensis) clearly trigger one or more of these pathways as a result of downstream events not reflected in the transcriptional responses, likely compromised phagolysosomal function or integrity, for which several explanations have been proposed (18, 20, 74, 75).

Brunke and coworkers recently described transcriptional interactions of *Candida* species inoculated into human blood, focusing on the most pathogenic species: C. albicans, C. parapsilosis, C. tropicalis, and C. glabrata (76). Similar to our observations, these authors also found highly similar host responses to different *Candida* species. In contrast, while they did identify some conserved fungal responses, including downregulation of ribosomal genes and an induced stress response, there was significantly greater species specificity, including in metabolic responses, than we show here. This is due in part to the different experimental systems between our isolated macrophages and the heterogeneous nature of whole blood. The other authors previously showed that the transcriptional response of C. albicans to blood is driven by neutrophil interactions (36), which induce a markedly different response in this species than do macrophages (17, 36). Additionally, while our study evaluates a range of species with wider virulence potential, it focuses on a single early time point (1 h) in comparison to the previous study that considered the first 4 h of interaction (76). We investigated early interactions to allow comparison of responses of fungal and macrophage cells under equivalent conditions since, at later time points, some species kill and escape the engulfing host cells whereas others do not, thus placing them under very different environmental conditions. Nevertheless, this represents a limitation of our study as other regulatory programs could emerge only later in the interaction. Overall, these two studies provide complementary views into how different *Candida* species interact with the host but underscore the complexity of these interactions and the range of environments to which invasive fungi must rapidly adapt.

This study represents the most extensive interspecies comparison of *Candida* transcriptomes including nonpathogenic species to date. Our transcriptional analysis has focused on single strains for each species, and given that significant intraspecies variation in transcriptional regulation has been observed (77), it will be important to determine how well the differences between species are consistent when different strains are compared. Nevertheless, what is most evident from these data is that, in contrast to regulation of hyphal morphogenesis, the response to phagocytosis is conserved across the virulence spectrum. While metabolic plasticity is important for virulence in C. albicans, rather than being an adaptation to a pathogenic lifestyle it is perhaps a key predisposing factor that underlies why this clade has been, and continues to be, such an important source of emerging opportunistic pathogens.

## MATERIALS AND METHODS

### *Candida* strains and media.

Details of *Candida* strains are provided in Table 3. Wild-type strains are genome reference strains (13, 78, 79) that were previously phenotypically characterized (33). Strains were routinely grown in YPD (1% yeast extract, 2% peptone, 2% dextrose). For RNA-seq and qRT-PCR experiments, strains were grown overnight at 30°C from YPD-agar plates (2% agar) and then diluted and allowed to return to log phase over at least 3 h in YPD at 30°C. For drop dilution tests, overnight YPD cultures were washed and then diluted to an optical density at 600 nm (OD_600_) of 1 in water. Fivefold dilutions were spotted on YNB-agar plates (0.17% yeast nitrogen base, 10 mM ammonium sulfate, 2% dextrose, 2% agar). For UV killing, C. albicans cultures in phosphate-buffered saline (PBS) were irradiated using a Spectroline ENB260C UV source (312 nm) at a distance of 1 cm for 15 min, resulting in at least 99.999% mortality (based on culture plating from three replicates).

**TABLE 3:** Details of *Candida* strains used in this study

Species	Strain	Full genotype	Origin of strain
Candida albicans	SC5314	Wild type	“Disseminated” (98)
Candida dubliniensis	CD36		Oral (99)
Candida tropicalis	MYA-3404		Blood (100)
Candida parapsilosis	CDC317		Skin (101)
	CDC317 + pNRVL	*NEUT5L*/*neut5l*::*pNRVL*	This study
	CDC317 + pCpHSP21	*NEUT5L*/*neut5l*::*pNRVL*::*PCpHSP21*::*HSP21*	This study
	CDC317 + pCpHSP21_400	*NEUT5L*/*neut5l*::*pNRVL*::*PCpHSP21_400*::*HSP21*	This study
	CDC317 + pCaHSP21	*NEUT5L*/*neut5l*::*pNRVL*::*PCaHSP21*::*HSP21*	This study
	CDC317 + pCpACT1	*NEUT5L*/*neut5l*::*pNRVL*::*PCpACT1*::*HSP21*	This study
*Lodderomyces elongisporus*	NRRL YB-4239		Orange juice (65)
Candida lusitaniae	ATCC 42720		Blood (102)

### Macrophage culture and media.

For fungus-enriched RNA-seq experiments, bone marrow-derived macrophages (BMDMs) were from homogenates of bone marrow from outbred female ICR mice, followed by differentiation *in vitro* with 20 ng/ml mouse granulocyte-macrophage colony-stimulating factor (GM-CSF) (R&D Systems) in Iscove’s modified Dulbecco’s medium (GE Healthcare) supplemented with 10% fetal bovine serum (Corning) and penicillin-streptomycin (Corning) (complete IMDM). For macrophage-enriched RNA-seq experiments, BMDMs were derived from female C57BL/6 mice and cultured with Dulbecco’s modified Eagle’s medium (high glucose; GE Healthcare) with 10% fetal bovine serum and penicillin-streptomycin (complete DMEM), differentiating with 10 ng/ml mouse M-CSF (BioLegend) for RNA-seq, qRT-PCR, and cytotoxicity assays and 20 ng/ml for ELISA and phagocytosis assays (due to batch variations). J774 cells (ATCC) were cultured in complete DMEM. All mammalian cell incubations were at 37°C, 5% CO_2_, and all mice were obtained from Envigo.

### RNA-seq sample preparation and sequencing.

For fungus-enriched RNA-seq experiments, BMDMs were obtained as described above and incubated for 24 h in complete IMDM supplemented with 20 ng/ml GM-CSF at 5 × 10^6^ cells per well in a 6-well plate. Log-phase *Candida* cultures were washed in phosphate-buffered saline (PBS) and then diluted into complete IMDM and added to wells in the presence or absence of BMDMs for 1 h at an MOI of 2, with two biological replicates. After incubation, *Candida*-only wells were scraped, pelleted by centrifugation at 1,000 × *g* for 5 min, and then resuspended in ice cold water and pelleted again at the same speed. *Candida*-macrophage cocultures were scraped in ice-cold water, pelleted by centrifugation at 1,000 × *g* for 5 min, washed in ice-cold water, and pelleted again at the same speed. To digest the fungal cell wall, samples were incubated for 5 min at 37°C with 40 U Zymolyase (United States Biological), followed by RNA extraction with the SV total RNA isolation system (Promega). For macrophage-enriched experiments, BMDMs from three mice were seeded overnight in 12-well plates (with 100 U/ml IFN-γ for the IFN-γ/LPS condition). Macrophages were stimulated with either *Candida* at an MOI of 3, complete DMEM alone, or medium supplemented with 100 U/ml IFN-γ and 100 ng/ml LPS for 1 h before medium was aspirated and RNA was extracted using the RNeasy Micro kit (Qiagen), which enriched for mouse RNA as it did not degrade the *Candida* cell wall. RNA integrity was monitored using a Bioanalyzer (Agilent Technologies). Libraries prepared with the TruSeq stranded RNA protocol (Illumina) were sequenced on a HiSeq 2000 (Illumina) to obtain 15 to 58 million 100-bp paired-end reads (fungal RNA) or on a NovaSeq 6000 (Illumina) to obtain 19 to 28 million 151-bp paired-end reads (macrophage RNA). Library preparation and sequencing were carried out by Psomagen, Inc.

### RNA-seq alignment and transcript quantification.

Quality control was performed using FastQC (https://www.bioinformatics.babraham.ac.uk/projects/fastqc/) and MultiQC (80). Adapters were trimmed using Trimmomatic v0.39 (81), with additional removal of bases below a read quality of 3 at the start and end of the read and total removal of reads less than 36 bp after trimming. For fungal RNA-seq, *Candida* genomes were obtained from the *Candida* Genome Database (http://www.candidagenome.org/) (42) and concatenated to the mouse genome (Ensembl release 98 [82]). For assessment of overall mapping efficiency, reads were aligned to the genome using STAR v2.7.3a (83), and mapping to each species was quantified by a custom Python script. For quantification of transcript abundance, a reference transcriptome was generated for each concatenated genome using GffRead v0.11.6 (84), followed by alignment-free quantification using Salmon v1.1.0 (85) with –gcBias and –seqBias flags to correct for fragment-level GC biases and sequence biases. Mouse transcripts were removed from Salmon output files prior to downstream analysis.

For macrophage RNA-seq experiments, STAR alignment was performed for all samples to a reference genome consisting of the mouse reference concatenated to all four *Candida* species used. Percent alignment was high (94 to 98%), with less than 1% of reads aligning to fungal genomes (see Fig. S1C in the supplemental material). Transcript quantification was performed using RSEM v1.3.1 (86), after which *Candida* transcripts were removed.

### Differential expression analysis and generation of abundance estimates of orthologous genes.

For fungal RNA-seq experiments, normalization and differential expression analysis were performed using DESeq2 v1.22.2 (87). For this, transcript abundance estimates from Salmon output were imported using the tximport package v1.10.1 (88). Pairwise differential expression analysis was done independently for each species. To get abundance estimates of orthologous genes across all species, 4,376 orthologous groups were identified using CGOB, and then custom R scripts were used to generate Salmon output (“quant.sf”) files for each sample containing all orthologs for all shared genes. For orthologs from species other than that of the sample itself, transcript abundance was set to zero and the real and effective transcript lengths were set to those of the *Candida*-only replicate 1 sample for the ortholog’s species of origin. This allowed transcript abundance to be estimated for each gene across species (i.e., for each group of orthologs) using tximport and DESeq2, while adjusting for differences in effective transcript length across orthologs (effectively, orthologs from different species are treated as alternative transcripts of the same gene, assigned the gene ID of C. albicans). Fragment counts were log-transformed using the regularized log_2_ transformation (rlog) function in DESeq2. For comparisons between C. parapsilosis and *L. elongisporus*, we included only these two species, which increased the number of orthologous groups of genes to 5,816, with orthologs assigned the gene ID of C. parapsilosis.

Macrophage gene-level abundance was estimated using the RNA-seq by Expectation Maximum (RSEM) approach with tximport, and differential expression analysis was performed using DESeq2 with log_2_ fold change shrinkage by the apeglm method (89). Differentially expressed genes were defined using an FDR of 0.1 and a LFC threshold of 1. Enrichment analysis of C. albicans-induced genes was performed using DAVID (53, 54) with a background of genes with at least 10 mapped reads across all samples (17,703 genes). *P* values were adjusted for multiple comparisons using the Benjamini-Hochberg procedure. Gene set enrichment analysis was performed using GSEA v4.0.3 (55, 56) on the same 17,703 expressed genes with the Hallmark gene sets v7.1 (90).

### Cluster analysis and comparison between C. parapsilosis and *L. elongisporus*.

Clustering was performed on root-mean-squared LFC estimates using the pam (partitioning around medoids) function in the R cluster package v2.1.0 (91). Cluster number (*k*) was selected using the “silhouette” method. GO term enrichment analysis was performed with the *Candida* Genome Database GO term finder tool with an FDR of 0.1 (42). To determine differentially induced genes between C. parapsilosis and *L. elongisporus*, expression, *E*, was modeled using DESeq2 as *E ∼* species + condition + species:condition, where condition is the presence or absence of macrophages and species:condition is the interaction between these two factors. Significant effects of the interaction term are equivalent to species-specific differences in the degree of phagocytosis-mediated induction. These were then intersected with genes identified as phagocytosis induced in pairwise comparison analyses to identify genes that were induced to a higher degree in one species than the other.

### qRT-PCR experiments.

For heat stress experiments, *Candida* in log phase was incubated in YPD at either 30°C or 42°C for 30 min. For J774 phagocytosis experiments, J774 cells were seeded overnight in complete DMEM, and then PBS-washed, log-phase *Candida* cultures were added at an MOI of 2 in complete DMEM to wells with and without J774 cells, coincubating for 1 h. RNA was isolated by hot acid phenol extraction, and genomic DNA was removed using Turbo DNase (Ambion). Gene expression was quantified using the Verso 1-step RT-qPCR kit (Thermo Fisher) with a CFX96 real-time PCR detection system (Bio-Rad), with *ACT1* used as the housekeeping gene in each species. For measurement of macrophage *Ccl3* expression, BMDMs were stimulated with *Candida* species at an MOI of 3 for 1 h, and then mouse RNA was isolated by TRIzol extraction (Ambion). qRT-PCR was performed as described above with *Gapdh* as the housekeeping gene, using previously described primers (*Gapdh* [92] and *Ccl3* [93]).

### C. parapsilosis strain construction.

Strains ectopically expressing *HSP21* (*CPAR2_209280*) were generated using the plasmid pNRVL-SAT1 (94) (a gift from Attila Gácser) using the NEBuilder HiFi DNA assembly kit (New England Biosciences). pNRVL-SAT1 was linearized using XhoI (New England Biosciences). For each strain, a plasmid was assembled from three fragments: the linearized pNRVL-SAT1 backbone, a promoter region, and the *HSP21* coding sequence (including 196 bp of downstream genomic sequence). Fragments were amplified using Phusion polymerase (New England Biosciences). Plasmids were linearized using StuI (New England Biosciences) for integration into the C. parapsilosis *NEUT5L* locus and then used to transform C. parapsilosis CDC317 by electroporation with 200-μg/ml nourseothricin selection (Jena Bioscience).

### Macrophage lysis assay.

Exponential-phase *Candida* in PBS was diluted into serum-free, phenol red-free RPMI (GE Healthcare) and added to macrophages at an MOI of 3 for 6 h before supernatants were taken and lactate dehydrogenase release was measured using the CytoTox nonradioactive cytotoxicity assay kit (Promega).

### Phagocytosis assay.

BMDMs were seeded overnight in 8-well chamber slides (Ibidi) before *Candida* was added at an MOI of 3. After 1 h, wells were washed and cells were fixed with 2.7% paraformaldehyde in PBS. Wells were then stained with Alexa Fluor 594-conjugated concanavalin A (10 μg/ml, 30 min, room temperature; Invitrogen) to stain extracellular fungal cells. Cells were then washed and permeabilized with Triton X-100 (0.1%, 3 min, room temperature; Sigma) and then subjected to blocking with 1% bovine serum albumin in PBS (30 min, room temperature). All fungal cells were then stained with fluorescein isothiocyanate (FITC)-conjugated anti-C. albicans rabbit polyclonal antibody (1,200× dilution, 4°C, overnight; LSBio). Nuclei were stained with NucBlue fixed cell stain (Invitrogen) in PBS, and images were taken using an IX83 inverted microscope with cellSens software (Olympus). The percentage of macrophages with at least one internalized fungal cell (green) as opposed to external (red and green) was quantified by counting at least 200 macrophages across three fields of view for each sample.

### Determination of cytokine secretion by ELISA.

Macrophages were stimulated with medium or with *Candida* at an MOI of 3 unless otherwise stated, and then cytokine concentration was determined from supernatant using either the mouse MIP-1 alpha (CCL3) or TNF-α uncoated ELISA kits (Invitrogen).

### Data visualization.

All figures were produced using the R package ggplot2 v3.2.1 (95) and assembled with Inkscape v1.0.0β2. Heatmaps were produced using the R function heatmap.3 (https://github.com/obigriffith/biostar-tutorials/blob/master/Heatmaps/heatmap.3.R).

### Data availability.

RNA-seq data sets have been deposited at the Gene Expression Omnibus (GEO) database with the accession numbers GSE151288 (fungal data sets) and GSE152700 (macrophage data sets). Code used for data analysis is available at https://github.com/andrewpountain/Candida-species.
