# Supplementary figures and images for: Interactions of Both Pathogenic and Nonpathogenic CUG Clade Candida Species with Macrophages Share a Conserved Transcriptional Landscape
Source: mBio. 2021 Dec 14;12(6):e03317-21. doi: 10.1128/mbio.03317-21 (PMC8669484; doi:10.1128/mbio.03317-21)

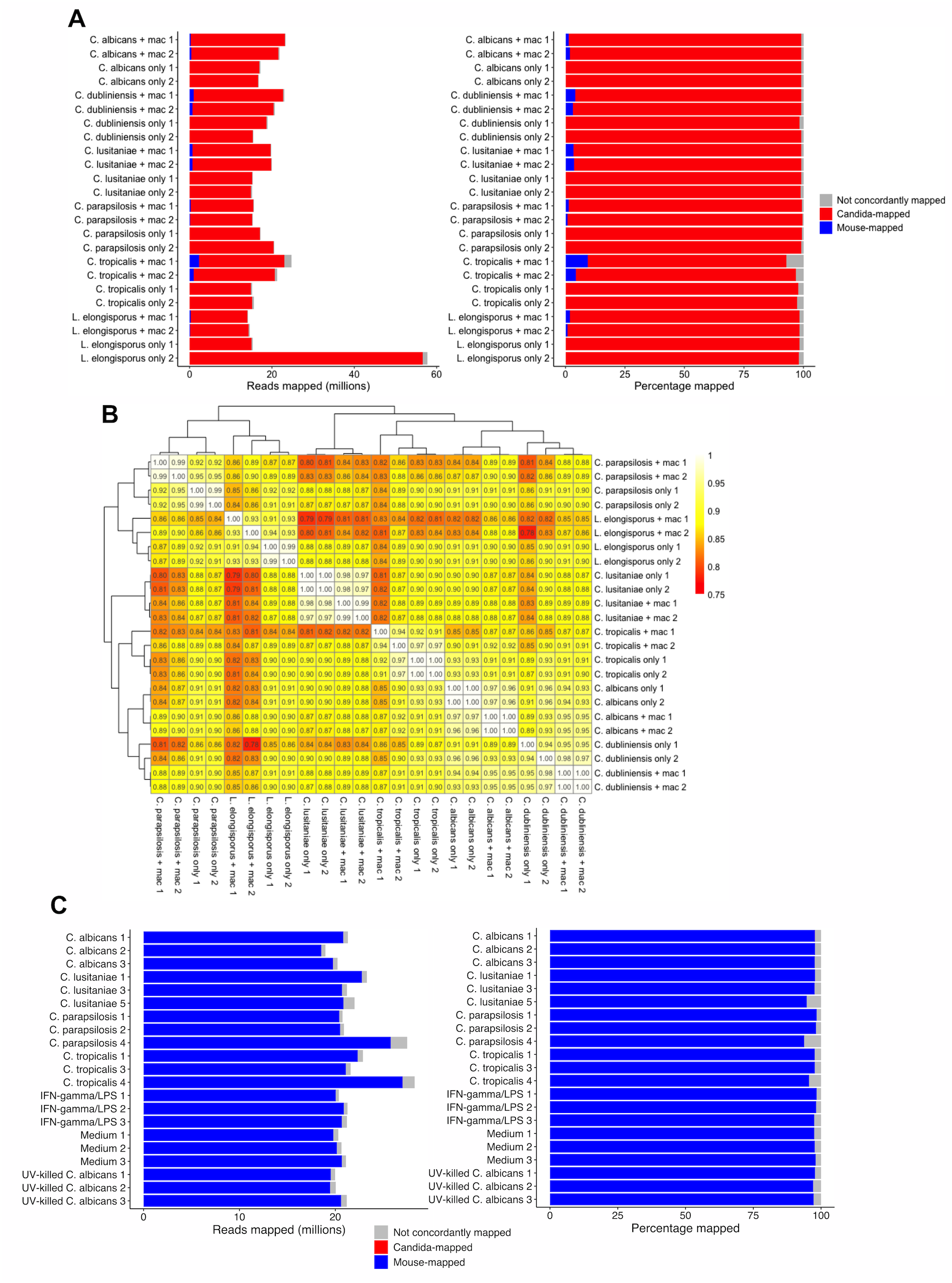

Supplement: FIG S1 [file mbio.03317-21-sf001.tif]

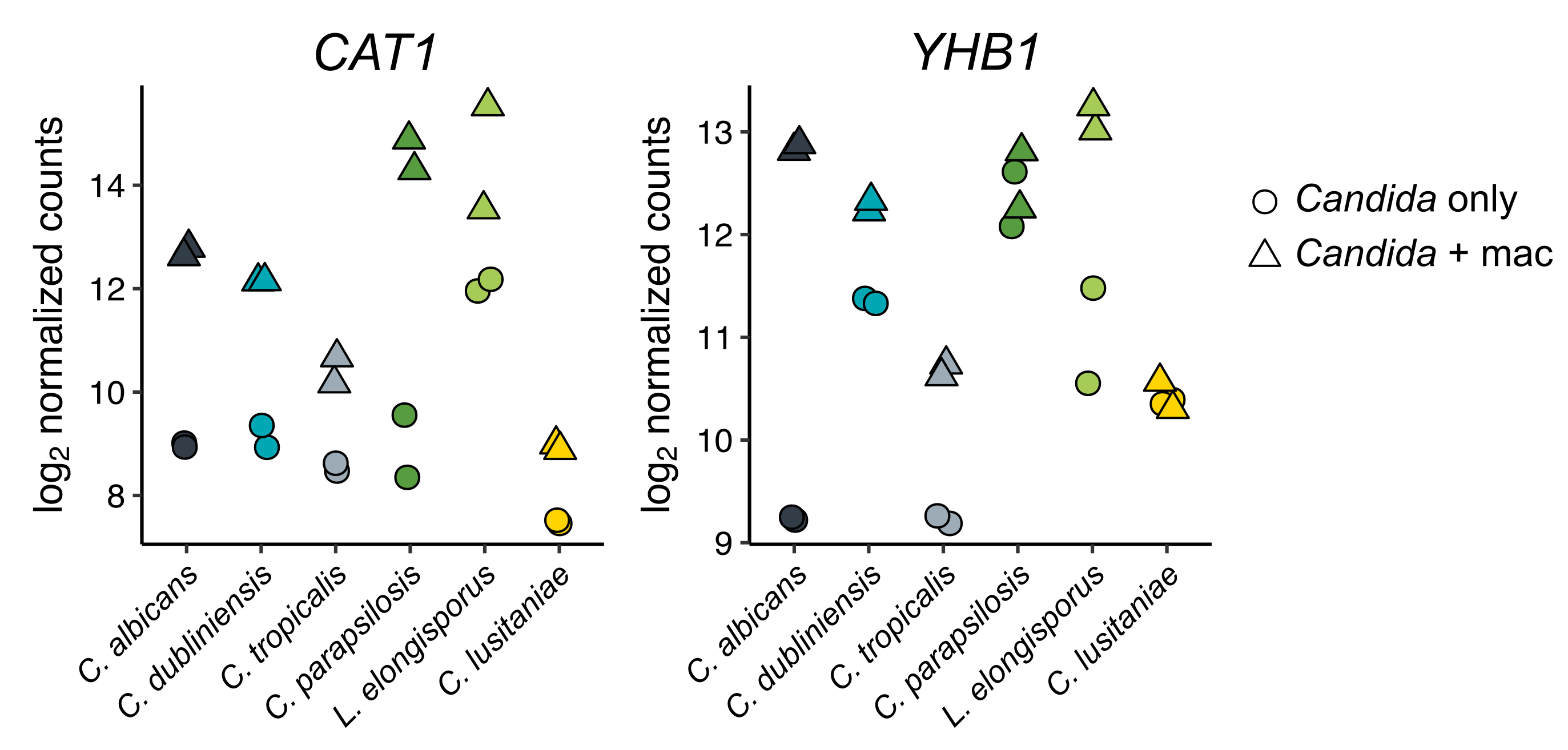

Supplement: FIG S2 [file mbio.03317-21-sf002.tif]

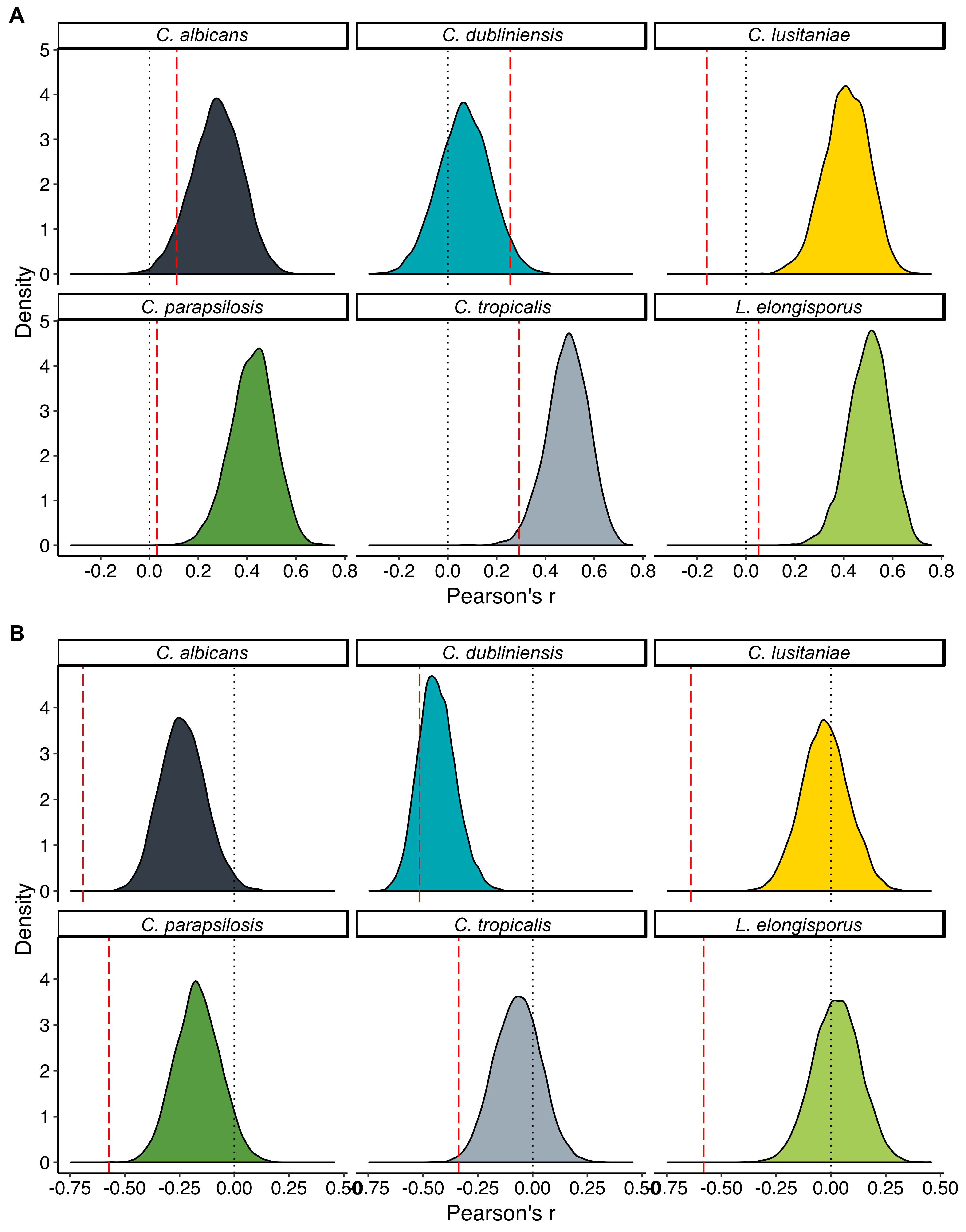

Supplement: FIG S3 [file mbio.03317-21-sf003.tif]

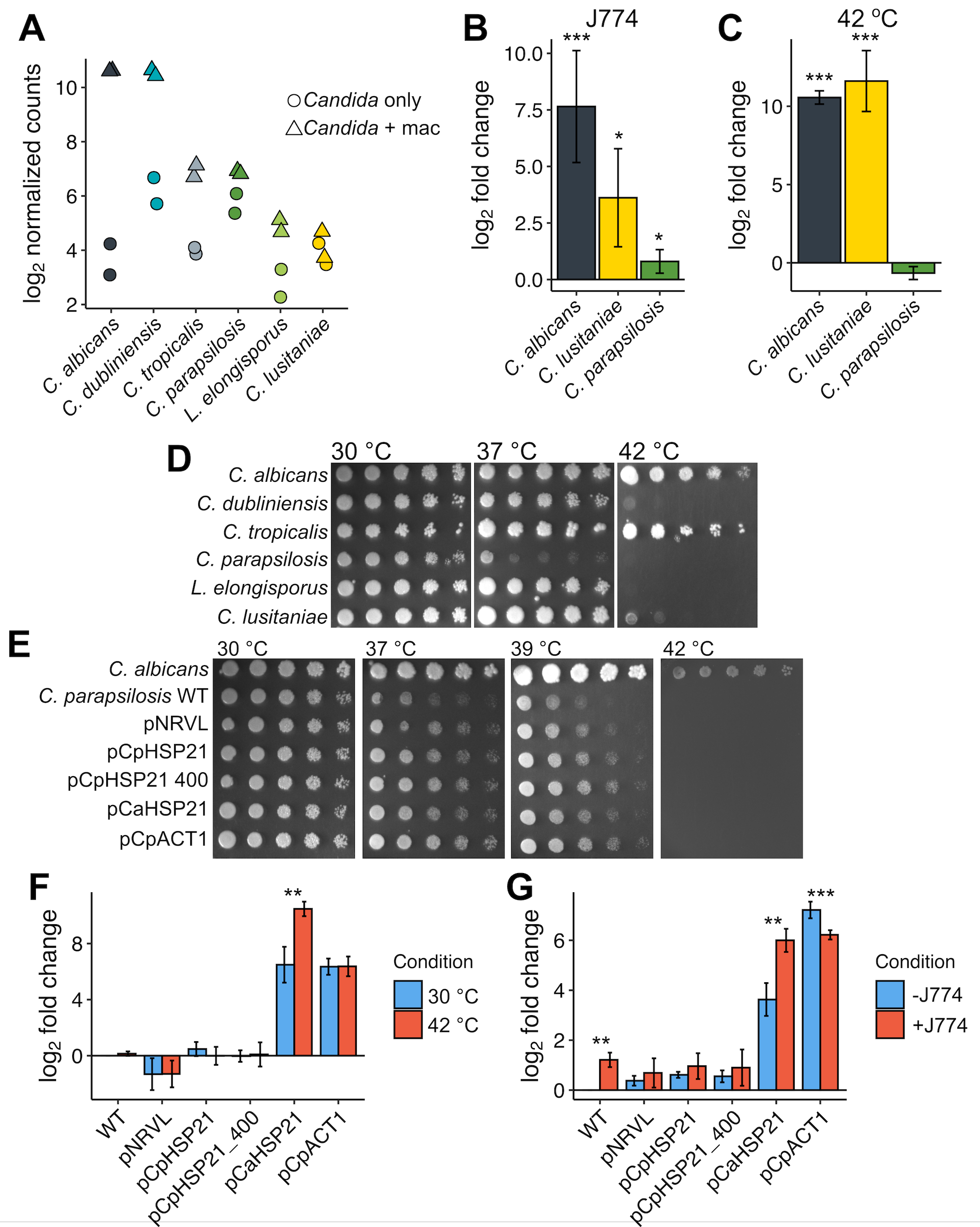

Supplement: FIG S4 [file mbio.03317-21-sf004.tif]

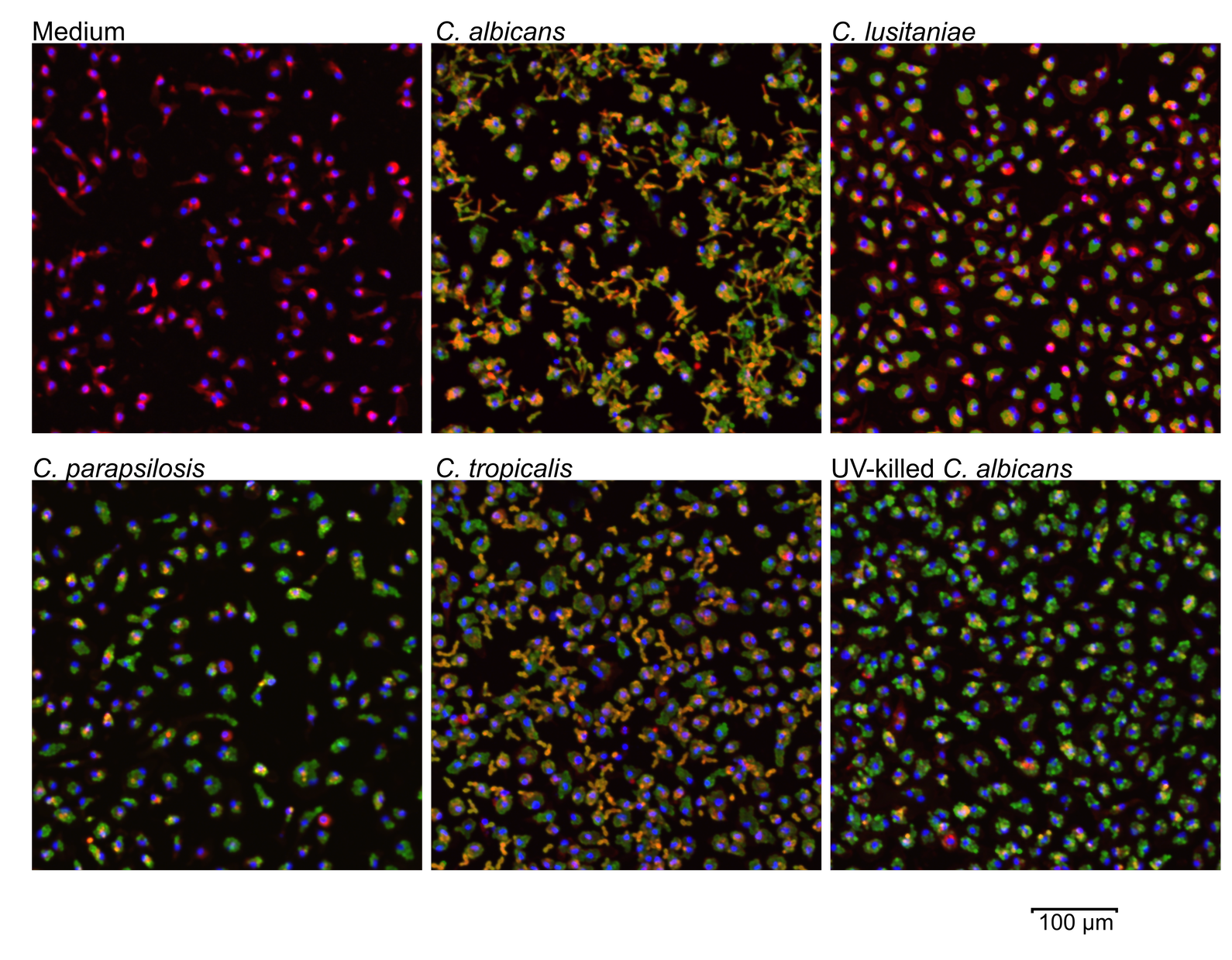

Supplement: FIG S5 [file mbio.03317-21-sf005.tif]

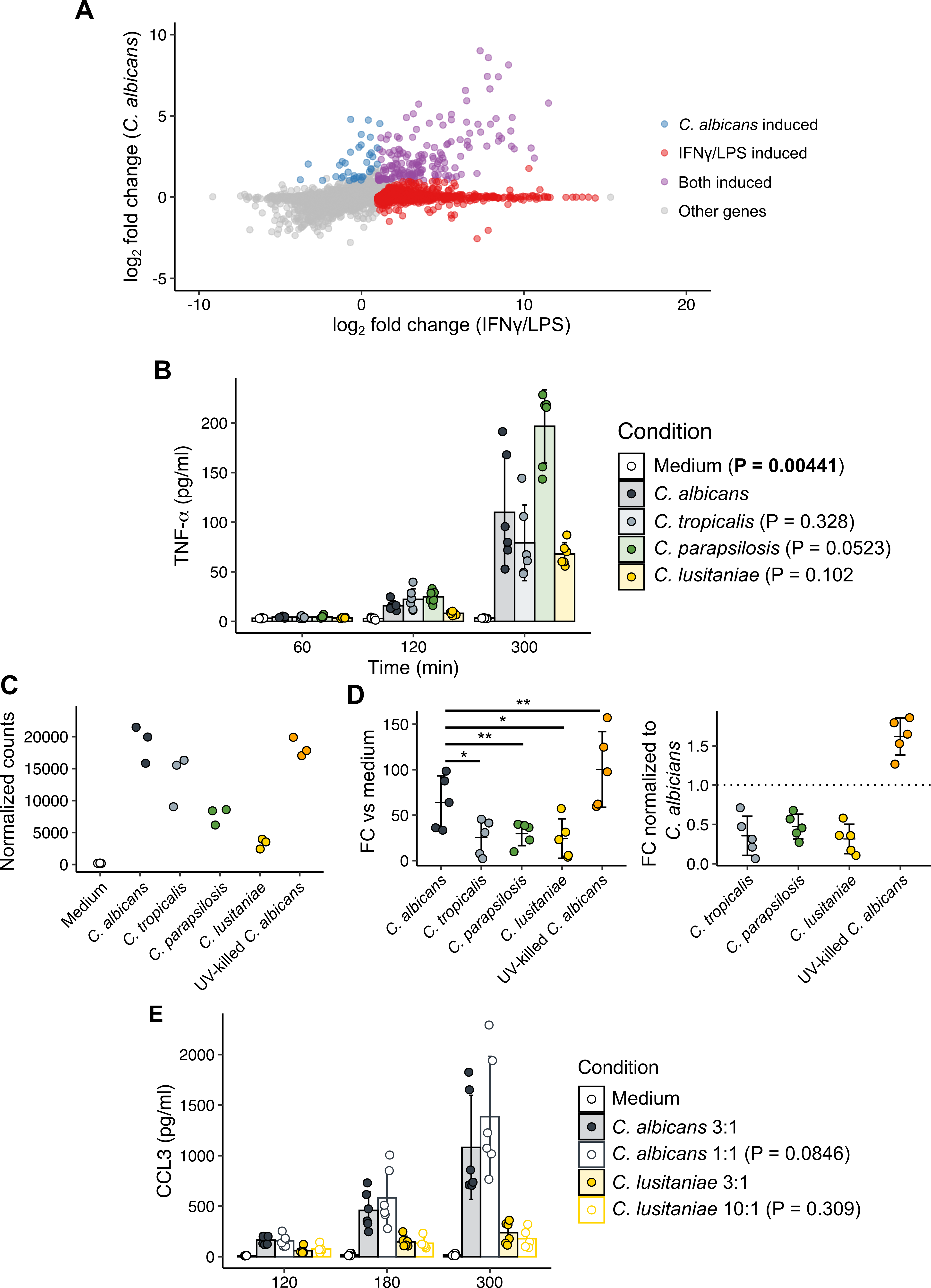

Supplement: FIG S6 [file mbio.03317-21-sf006.tif]
